# Behavioral and Biochemical Effects of Vitexin–Isovitexin-Loaded Microcapsule Combination on Restraint Stress Mouse Model

**DOI:** 10.3390/ijms27198731

**Published:** 2026-09-30

**Authors:** Huynh Nhu Mai, Kien Duc Ngo, Manh Hung Tran, Duc Tuan Nguyen, Hoang-Han Do, Lam Truong Tuong, Viet-Cuong Nguyen, Nopphadol Udomluck, Phuong Tram Nguyen, Minh Hien Khuu, Ngoc Phuc Nguyen Nguyen, Nhu Y Vo, Minh-Quan Le

**Affiliations:** 1 School of Pharmacy, University of Medicine and Pharmacy at Ho Chi Minh City, Ho Chi Minh City 70000, Vietnam; mhnhu@ump.edu.vn (H.N.M.); ngokienduc@ump.edu.vn (K.D.N.); manhhung@ump.edu.vn (M.H.T.); ductuan@ump.edu.vn (D.T.N.); dhhan@ump.edu.vn (H.-H.D.); tuonglamtruong@ump.edu.vn (L.T.T.); khuuminhhien6688@gmail.com (M.H.K.); nguyenngocphucnguyen2003@gmail.com (N.P.N.N.); vnhuy.duoc22@ump.edu.vn (N.Y.V.); 2 Faculty of Pharmacy, Lac Hong University, Dong Nai 76000, Vietnam; vietcuong@lhu.edu.vn; 3 Department of Pharmaceutical Sciences, Faculty of Pharmacy, Chiang Mai University, Chiang Mai 50200, Thailand; n.udomluck@gmail.com; 4 Research Group in Pharmaceutical and Biomedical Sciences, Faculty of Pharmacy, Ton Duc Thang University, Ho Chi Minh City 70000, Vietnam; nguyenphuongtram@tdtu.edu.vn; 5 Faculty of Sciences, University of Montpellier, 34000 Montpellier, France

**Keywords:** vitexin–isovitexin, restraint stress, cortisol, microencapsulation, HPA axis

## Abstract

Chronic stress and anxiety highlight the need for safer natural alternatives to conventional therapies with severe adverse effects, yet the combined behavioral and biochemical effects of vitexin and its structural isomer, isovitexin, remain insufficiently investigated. This study evaluated the effects of a vitexin–isovitexin mixture in both crude and microencapsulated forms under acute and repeated restraint stress. In vitro radical-scavenging activity was assessed via the DPPH assay. In vivo, male Swiss albino mice subjected to acute (4 h) and repeated (4 h × 7 days) restraint stress received oral administration of the mixture (30 mg/kg) 30 min before each stress session, followed by behavioral testing and biochemical analysis; molecular docking was used as an exploratory approach to assess predicted structural compatibility with selected stress-related targets. In vitro, the mixture exhibited DPPH radical-scavenging activity. In vivo, the effects differed between the acute and repeated stress models, with significant changes in several behavioral outcomes, serum cortisol, and GSH levels, whereas MDA and TST responses were less consistent. Molecular docking suggested predicted structural compatibility of vitexin and isovitexin with SERT, 5-HT1A, GABAA, the glucocorticoid receptor, and Keap1. Overall, the VTX-iVTX mixture showed variable effects on stress-related behavioral and biochemical outcomes, with microencapsulation showing effects comparable to the crude form in most parameters and more pronounced effects in selected outcomes.

## 1. Introduction

In modern society, chronic stress and prolonged anxiety are considered major risk factors contributing to the increasing prevalence of mental health disorders. According to the World Health Organization (WHO), in 2025, anxiety and depression contributed substantially to the global disease burden and years lived with disability [1]. Following the COVID-19 pandemic in 2020, the global prevalence of anxiety disorders increased by approximately 25% compared with the previous year [2]. Stress has also been recognized as a major contributor to physical and mental health problems, including cardiovascular disease, Alzheimer’s disease, and certain cancers [3].

Anxiety and depressive disorders are associated with disturbances in neurotransmitter systems, including cholinergic, catecholaminergic, and serotonergic signaling, especially 5-hydroxytryptamine (5-HT), as well as alterations in neuroendocrine regulation, particularly within the hypothalamic–pituitary–adrenal (HPA) axis [4]. Based on these mechanisms, antidepressant medications such as selective serotonin reuptake inhibitors (SSRIs), tricyclic antidepressants (TCAs), and monoamine oxidase inhibitors (MAOIs) have been widely prescribed. However, these therapies may cause serious adverse effects and often require long-term treatment to maintain symptom remission [5,6]. Considering these limitations, herbal pharmaceutical approaches are being increasingly investigated as potential alternatives.

Vitexin and isovitexin are flavonoid compounds frequently found together in various medicinal plants, including pigeon pea, passiflora, and mung bean seed coat. They are structural isomers and mono-C-glycoside derivatives of apigenin, sharing similar chemical properties and displaying antioxidant and health-protective effects (Figure S1). Vitexin and isovitexin display stability along with antioxidant [7,8,9]. However, research on their neurological effects remains limited. To date, only one in vivo study has evaluated the antidepressant-like effect of vitexin, showing reduced immobility time in tail suspension and modified forced swimming tests and suggesting a monoaminergic mechanism [10]. Moreover, existing studies have mainly focused on purified vitexin and overlooked its mixture with isovitexin as commonly found in herbal extracts. We previously reported the bioavailability [11] and hypoglycemic effects [11] of this microencapsulated formulation. Nevertheless, the effects of this fixed vitexin–isovitexin mixture under stress conditions remain unclear.

Furthermore, the clinical application of vitexin and isovitexin is limited by their poor oral bioavailability [10]. Therefore, several novel drug delivery systems have been explored to improve the pharmaceutical performance of vitexin, including liposomes [12], microemulsions [13], β-cyclodextrin-based microspheres [14] and nano- or micro-scale particles [14,15]. However, the application of a microencapsulated formulation specifically designed to improve the delivery of the vitexin–isovitexin mixture under different stress conditions remains to be fully established.

Various rodent models have been developed to investigate stress-related behavioral and neurobiological changes, including restraint stress [16], chronic unpredictable mild stress (CUMS) [17], social isolation [4], and social defeat stress (SDS) models [18]. The restraint stress model involves placing a mouse in a confined apparatus that restricts spontaneous movement for a defined period and can be applied in both acute [19] and repeated [20] paradigms. This model provides standardized and controlled stress exposure and has been used to investigate stress-related neuroendocrine, behavioral, and inflammatory responses [21]. The model is commonly assessed based on behavioral criteria such as reduction in exploratory activity changes in exploratory activity, anxiety-related behavior, and passive coping behavior [22]. In addition, this model offers practical advantages including straightforward implementation, shorter experimental duration and cost efficiency.

The application of restraint stress models, combined with evidence supporting the biological activities of vitexin and isovitexin, provides a foundation for further preclinical studies. However, the behavioral and biochemical effects of the vitexin–isovitexin combination under restraint stress remain insufficiently studied. To overcome the pharmacokinetic limitations of these compounds, microencapsulation offers a delivery strategy to improve their stability and oral bioavailability. Accordingly, this study evaluates both the crude and microencapsulated forms of the vitexin–isovitexin mixture in acute restraint stress (ARS) and repeated restraint stress (RRS) models, focusing on behavioral and biochemical outcomes. Molecular docking was additionally used as an exploratory approach to examine the predicted structural compatibility of vitexin and isovitexin with selected stress-related molecular targets, while the role of microencapsulation was evaluated separately as a drug delivery strategy.

## 2. Results

### 2.1. In Vitro Antioxidant Activity of Vitexin–Isovitexin-Loaded Microcapsules via DPPH Free Radical Scavenging Assay

**Figure 1:**
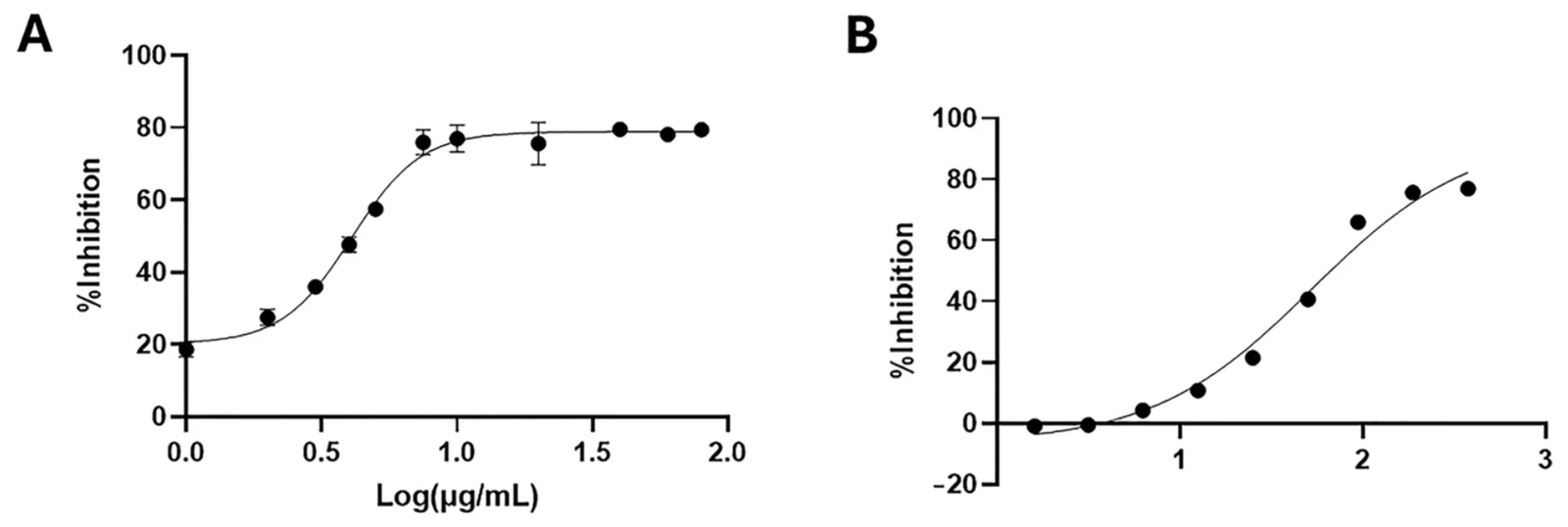
Free radical scavenging activity of the positive control and test samples using DPPH assay. (**A**) Ascorbic acid; (**B**) VTX-iVTX. Data are expressed as mean ± S.D. (*n* = 3).

The IC_50_ values of ascorbic acid and vitexin–isovitexin (VTX-iVTX) were 4.028 μg/mL (Figure 1A) and 52.50 μg/mL (Figure 1B), respectively. The antioxidant property of VTX-iVTX extract was 13.02 times lower than that of ascorbic acid.

### 2.2. Effects of Vitexin–Isovitexin-Loaded Microcapsules on Malondialdehyde (MDA) Levels

**Figure 2:**
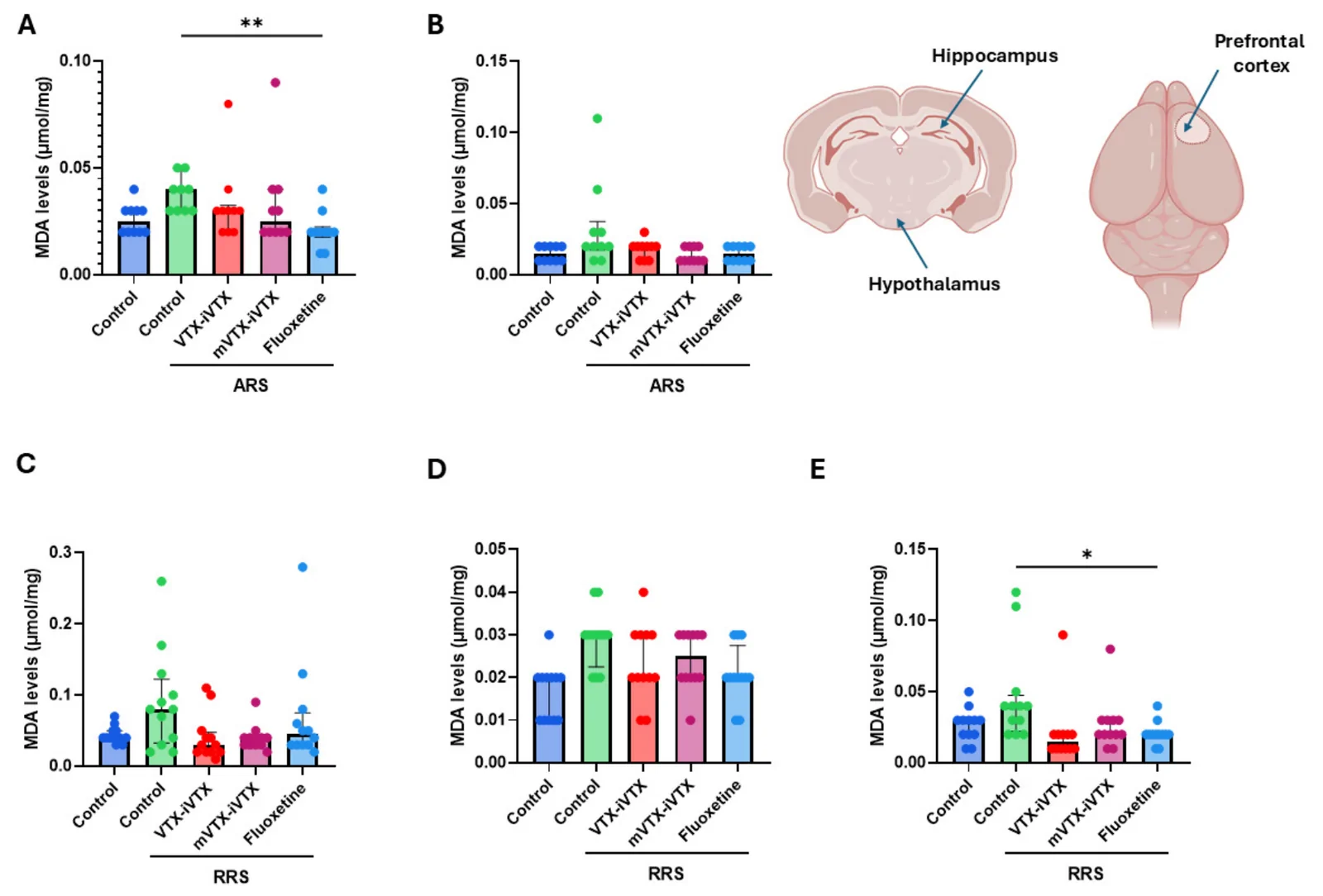
Malondialdehyde (MDA) levels (μmol/mg) in the hippocampus (**A**) and hypothalamus (**B**) in the ARS model, and in the hippocampus (**C**), hypothalamus (**D**), and prefrontal cortex (**E**) in the RRS model. Data are expressed as mean ± S.E.M. or median with interquartile range (IQR). ARS: *n* = 10 mice/group; RRS: *n* = 12 mice/group. Statistical analysis was performed using Kruskal–Wallis test followed by Dunn’s post hoc test. * *p* < 0.05, ** *p* < 0.01. ARS, acute restraint stress; RRS, repeated restraint stress. Created in BioRender. at Ho Chi Minh City, U. O. M. A. P. (2026) https://BioRender.com/5ej944z.

Following 4-h restraint, MDA levels in both the hippocampus (Figure 2A) and hypothalamus (Figure 2B) of the stress control group increased compared with those in the normal control group, but no statistically significant difference was observed. In the hippocampus, fluoxetine treatment significantly reduced MDA levels compared with the stress control group (*p* = 0.0012), whereas no other treatment groups reached statistical significance in this region (*p* > 0.05). In the hypothalamus, MDA concentrations were numerically lower across all treatment groups compared with the stress control group, but none of these reductions reached statistical significance.

In parallel, following a 7-day prolonged stress induction, a non-significant elevation in MDA levels was observed across three brain regions (Figure 2C–E) compared with the normal control group. Conversely, mice treated with VTX-iVTX, mVTX-iVTX, and fluoxetine exhibited a declining trend in hippocampal and hypothalamic MDA levels compared with the stress control group. Only in the prefrontal cortex did the fluoxetine group achieve a statistically significant decrease in MDA concentrations (Figure 2E, 55% reduction, *p* = 0.0401). Notably, no statistically significant differences were observed between the treatment groups themselves (*p* > 0.05).

### 2.3. Effects of Vitexin–Isovitexin-Loaded Microcapsules on Glutathione (GSH) Levels

**Figure 3:**
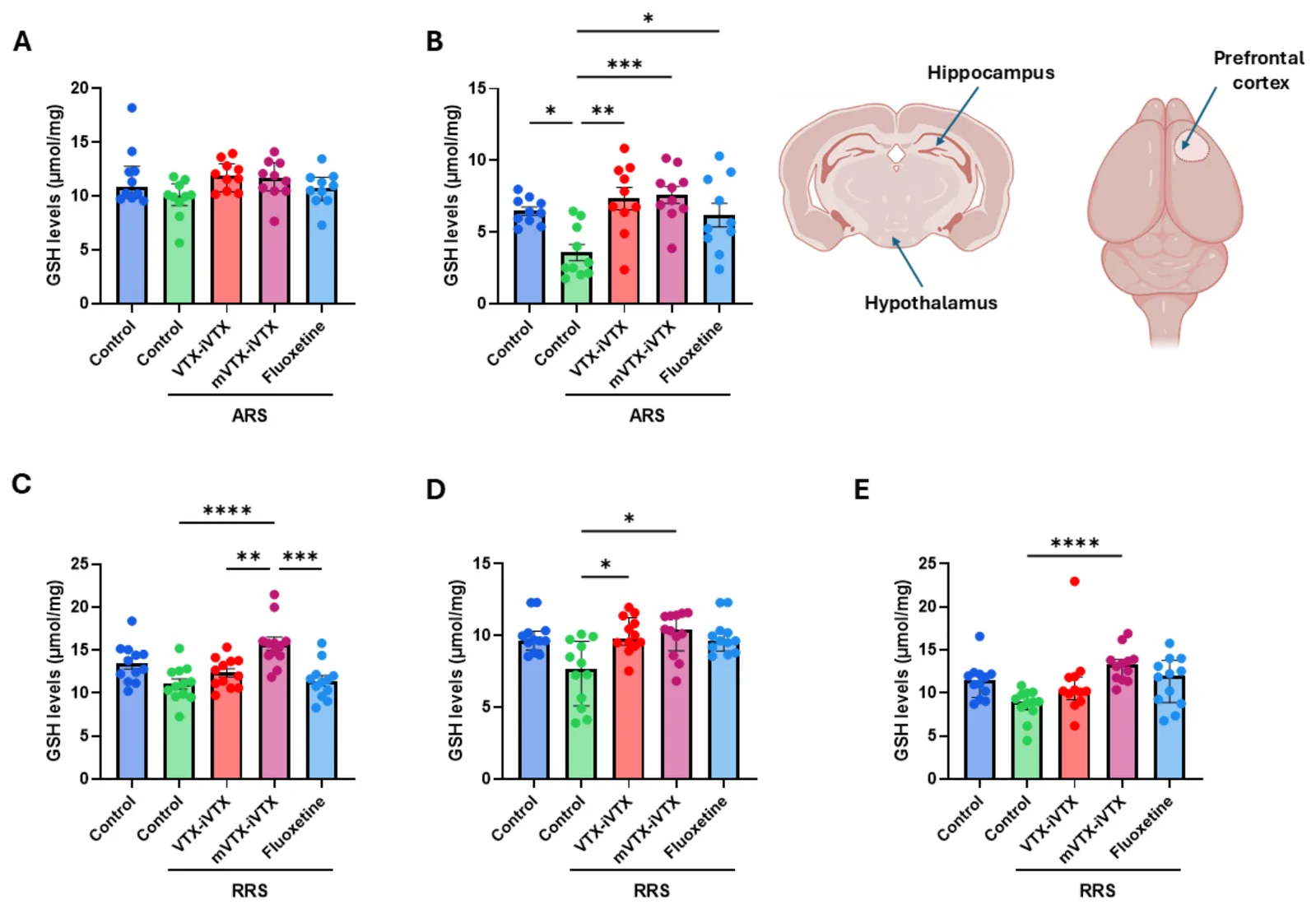
Glutathione (GSH) levels (μmol/mg) in the hippocampus (**A**) and hypothalamus (**B**) in the ARS model, and in the hippocampus (**C**), hypothalamus (**D**), and prefrontal cortex (**E**) in the RRS model. Data are expressed as mean ± S.E.M. or median with interquartile range (IQR). ARS: *n* = 10 mice/group; RRS: *n* = 12 mice/group. Statistical analysis was performed using one-way ANOVA followed by Tukey’s post hoc test (**B**,**C**) or Kruskal–Wallis test followed by Dunn’s post hoc test (**A**,**D**,**E**). * *p* < 0.05, ** *p* < 0.01, *** *p* < 0.001, and **** *p* < 0.0001. ARS, acute restraint stress; RRS, repeated restraint stress. Created in BioRender. at Ho Chi Minh City, U. O. M. A. P. (2026) https://BioRender.com/5ej944z.

Acute stress induced a decrease in GSH levels in both brain regions. Specifically, in the hypothalamus (Figure 3B), GSH concentrations in the stress control group exhibited a statistically significant decrease compared to the normal control group (*p* = 0.0195). While all treated groups produced a slight recovery in hippocampal GSH concentrations (Figure 3A), these alterations did not reach statistical significance (*p* > 0.05). In contrast, hypothalamic (Figure 3B) GSH concentrations remarkably increased by 2.1-fold after administration of both formulations compared to the stress control group (crude form, *p* = 0.0012 and microencapsulated form, *p* = 0.0005), with no statistically significant difference between two groups.

With respect to the RRS model, the stress control group demonstrated a decrease in GSH levels across all brain regions including the hippocampus (Figure 3C), hypothalamus (Figure 3D), and prefrontal cortex (Figure 3E) compared to the normal control group, although these reductions lacked statistical significance (*p* > 0.05). Following 7-day administration, mVTX-iVTX induced a statistically significant increase in GSH levels across all regions compared with the stress control group: hippocampus (42%, *p* < 0.0001), hypothalamus (37%, *p* = 0.0111), and prefrontal cortex (53%, *p* < 0.0001). In contrast, VTX-iVTX treatment significantly increased GSH levels only in the hypothalamus (*p* = 0.0381) while the increases observed in the hippocampus and prefrontal cortex did not reach statistical significance. Fluoxetine treatment also increased GSH levels across all three regions; however, these changes did not reach statistical significance. Notably, in the hippocampus, GSH levels in the mVTX-iVTX group were significantly higher than those in both the VTX-iVTX and fluoxetine groups (*p* = 0.0034 and *p* = 0.0001, respectively).

### 2.4. Effects of Vitexin–Isovitexin-Loaded Microcapsules on Serum Cortisol Levels

**Figure 4:**
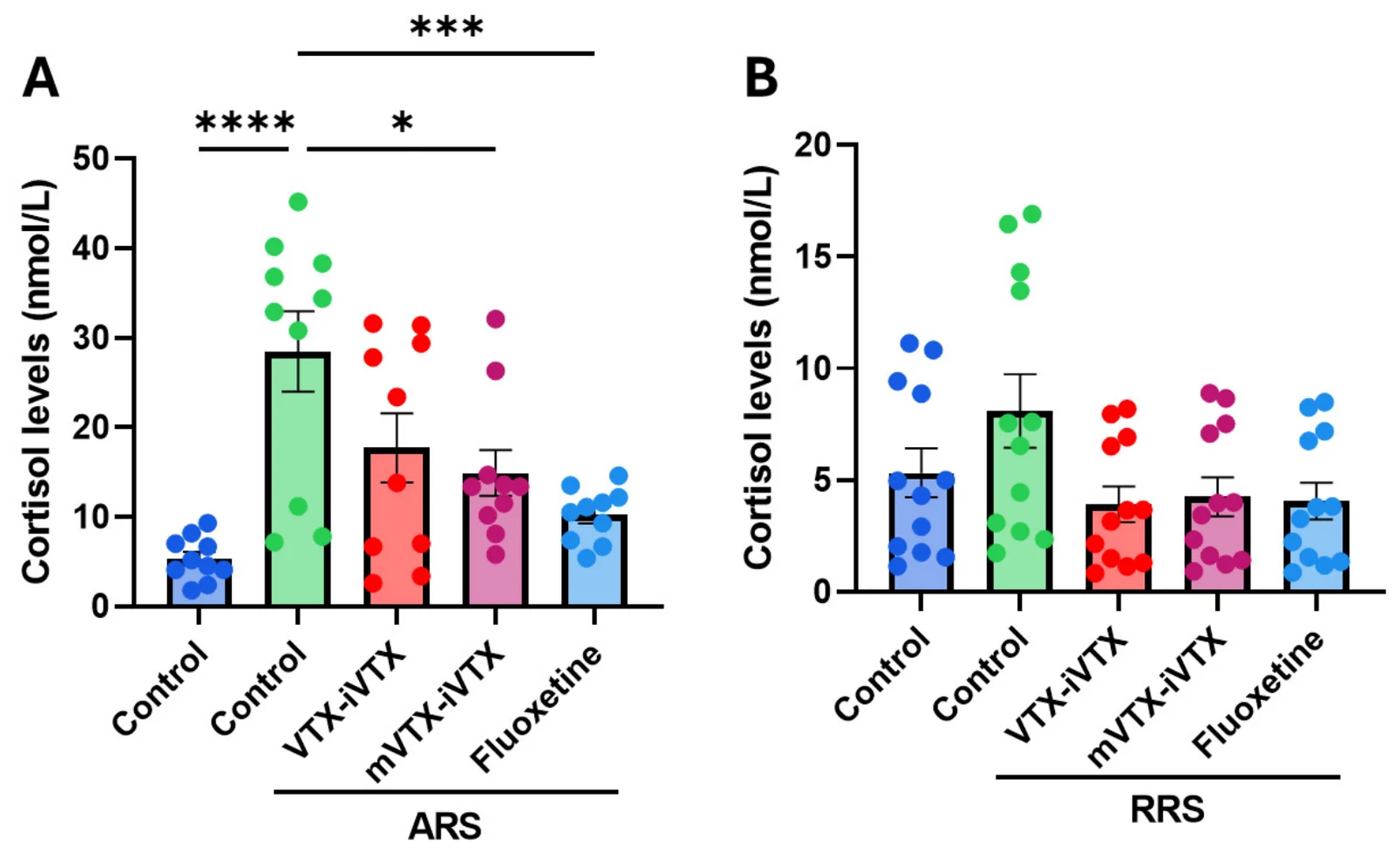
Serum cortisol levels (nmol/L) in the ARS (**A**) and RRS (**B**) model. Data are expressed as mean ± S.E.M. or median with interquartile range (IQR). ARS: *n* = 10 mice/group; RRS: *n* = 12 mice/group. Statistical analysis was performed using one-way ANOVA followed by Tukey’s post hoc test. * *p* < 0.05, *** *p* < 0.001, and **** *p* < 0.0001. ARS, acute restraint stress; RRS, repeated restraint stress.

As shown in Figure 4A, the stress control group marked an approximately fivefold elevation in cortisol levels relative to the normal control group (*p* < 0.0001). Administration of both formulations attenuated cortisol response to stress, with 38% reduction for the crude form and 48% reduction for the microencapsulation. However, only the microencapsulated formulation reached a statistically significant reduction (*p* = 0.0170). Similarly, the fluoxetine group showed a 64% decrease in cortisol levels in comparison with the stress control group (*p* = 0.0006).

Despite no statistically significant difference between the normal and stress control groups, an increase was recorded after 7-day stress exposure (Figure 4B). Conversely, treated mice diminished cortisol levels by half relative to the stress control group, with the crude form showed the most significant decrease (52% reduction), followed by fluoxetine (48%) and mVTX-iVTX (47%). However, none of these reductions reached statistical significance, and no significant difference was observed between the treatment groups.

### 2.5. Effects of Vitexin–Isovitexin-Loaded Microcapsules on the Novel Object Recognition (NOR) Test

**Figure 5:**
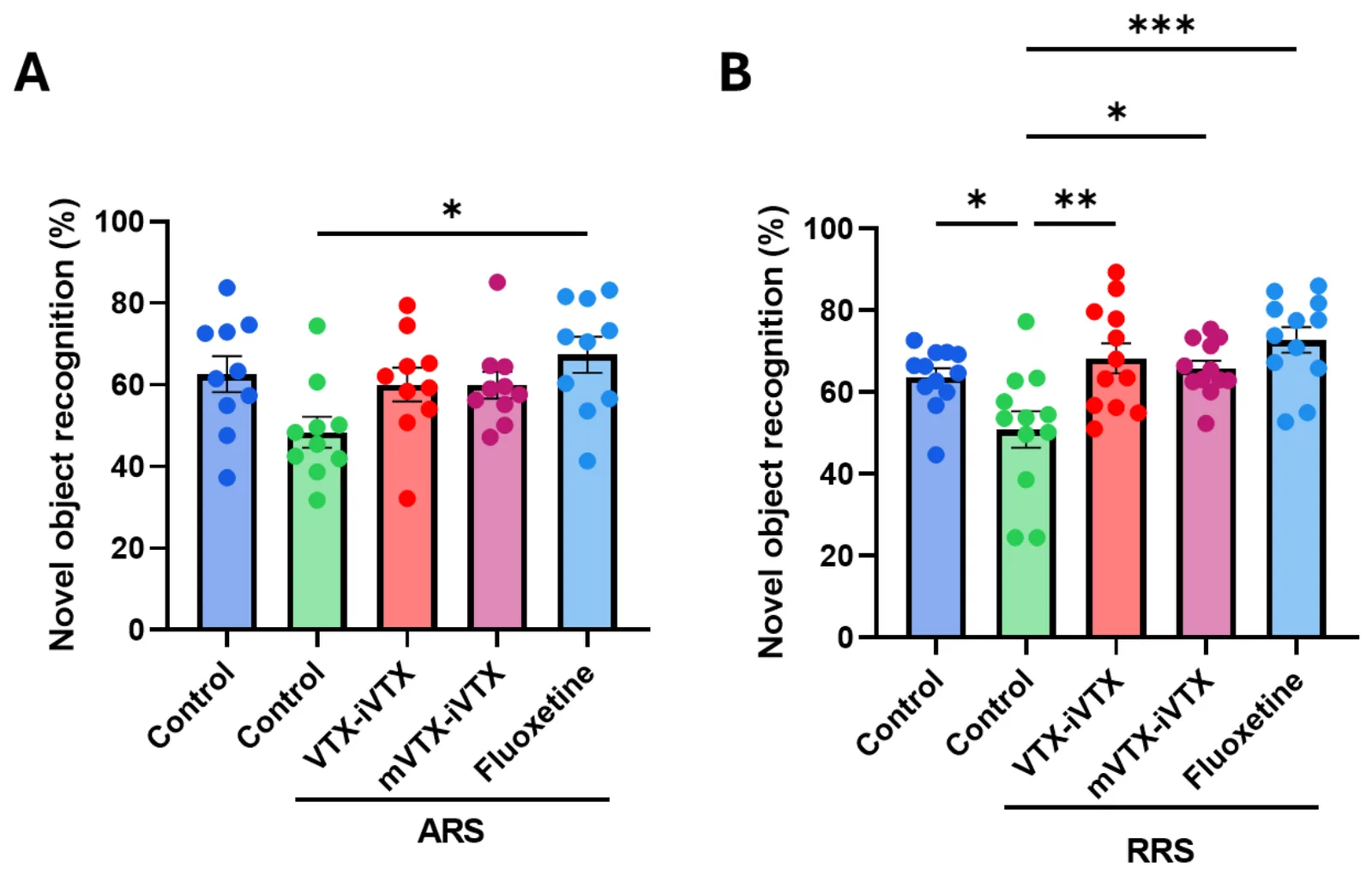
Novel object recognition (NOR) ratio (%) in ARS (**A**) and RRS (**B**) model. Data are expressed as mean ± S.E.M. or median with interquartile range (IQR). ARS: *n* = 10 mice/group; RRS: *n* = 12 mice/group. Statistical analysis was performed using Kruskal–Wallis test followed by Dunn’s post hoc test. * p < 0.05, ** *p* < 0.01, and *** *p* < 0.001. ARS, acute restraint stress; RRS, repeated restraint stress.

Figure 5A demonstrates the percentage of novel object recognition in the acute stress model. A 1.29-fold reduction in this ratio was observed in the stress control group compared to the normal control group, but no statistically significant difference was observed. In contrast, all treated groups displayed a higher novel object exploration ratio than that of the stress control group. Mice treated with VTX-iVTX and mVTX-iVTX both showed an approximately 1.24-fold increase in comparison with the stress control group (*p* > 0.05), while only the 1.39-fold increase in the fluoxetine group achieved statistical significance (*p* = 0.0143). Nevertheless, there was no statistical difference between these treatment groups.

It could be seen in Figure 5B that the ratio of time recognizing novel objects of the stress control group was 20% lower than that of the normal control (*p* = 0.0499) after 7-day stress exposure. In contrast, a significant increase was observed in VTX-iVTX (34%, *p* = 0.0030), mVTX-iVTX (29%, *p* = 0.0149), and fluoxetine (43%, *p* = 0.0001) groups, with no significant difference in the exploration ratio among these treated groups.

### 2.6. Effects of Vitexin–Isovitexin-Loaded Microcapsules on Social Interaction (SI) Test

**Figure 6:**
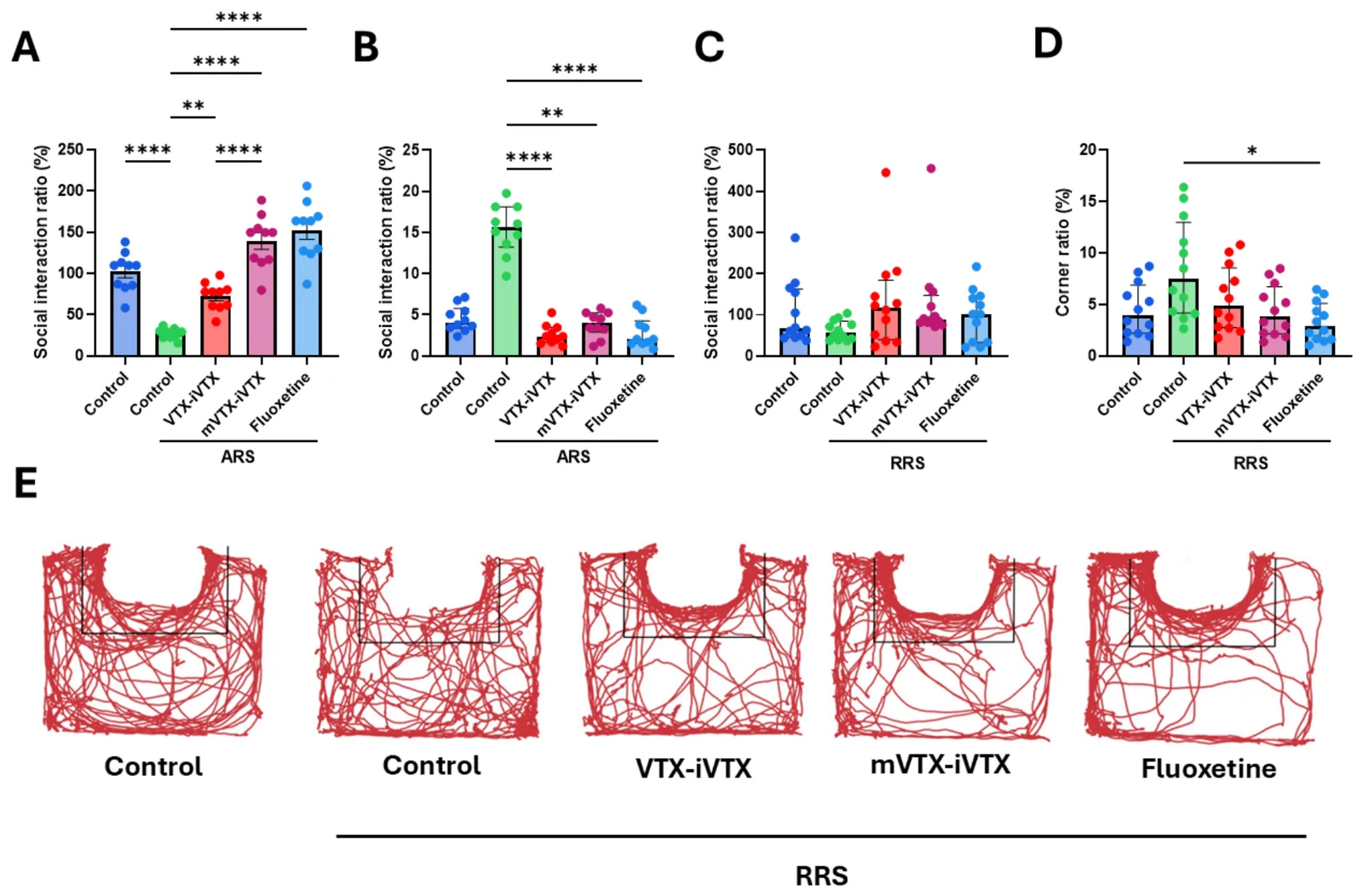
Social interaction ratio (**A**) and corner ratio (**B**) in the ARS model; social interaction ratio (**C**) and corner ratio (**D**) in the RRS model. Representative trajectory track maps of movement patterns in the arena during the RRS exposure using Toxtrac software (**E**). Data are expressed as mean ± S.E.M. or median with interquartile range (IQR). ARS: *n* = 10 mice/group; RRS: *n* = 12 mice/group. Statistical analysis was performed using one-way ANOVA followed by Tukey’s post hoc test (**A**) or Kruskal–Wallis test followed by Dunn’s post hoc test (**B**–**D**). * *p* < 0.05, ** *p* < 0.01, **** *p* < 0.0001. ARS, acute restraint stress; RRS, repeated restraint stress.

As seen in Figure 6A, the stress control group showed a significant reduction in the percentage of time spent in the interaction zone compared to the normal control group (*p* < 0.0001). In the treatment groups, the social interaction ratio was remarkably higher than in the stress control group. Compared with the stress control group, fluoxetine and mVTX-iVTX produced 5.61-fold and 5.14-fold increases, respectively (*p* < 0.0001 for both), followed by VTX-iVTX with a 2.66-fold increase (*p* = 0.0018). Importantly, both fluoxetine and mVTX-iVTX significantly outperformed VTX-iVTX (*p* < 0.0001). Conversely, the percentage of time spent in the corner zone was markedly increased in the stress control group, though without statistical significance (Figure 6B). All treatment groups showed a significant reduction in the corner ratio compared to the stress control group. While this reduction reached statistical significance in the VTX-iVTX and fluoxetine groups (both approximately 82% reduction, *p* < 0.0001), it was less significant in the mVTX-iVTX group (75% reduction, *p* = 0.0063).

After 7 days of stress induction, mice in the stress control group spent less time interacting with the novel mice (Figure 6C) compared to the normal control group, despite no statistically significant difference between two groups. While all treatment groups showed an increase in interaction ratio in comparison with the stress control group, these numerical changes were not statistically significant. Contrasting with social interaction behavior, the stress control group showed a higher percentage of time spending in the corner zone (Figure 6D) in comparison with the normal control group, but no statistically significance was observed. Consistently, VTX-iVTX and mVTX-iVTX groups showed a 34% and 48% reduction respectively, though these reductions did not reach statistical significance. Meanwhile, the fluoxetine group exhibited a more remarkable percentage with a decrease of approximately 61% (*p* = 0.0148). However, there was no statistically significant difference among these therapeutic groups. In addition, the mouse locomotor was recorded, with Figure 6E illustrating the patterns during the SI test in the RRS model. Mice in the stress control group showed a distinct social avoidance pattern, characterized by corner occupancy. All treatment groups showed frequent transitions between the corner and interaction zones, with an exploratory preference for the interaction zone rather than the corner one.

### 2.7. Effects of Vitexin–Isovitexin-Loaded Microcapsules on Tail Suspension Test (TST)

**Figure 7:**
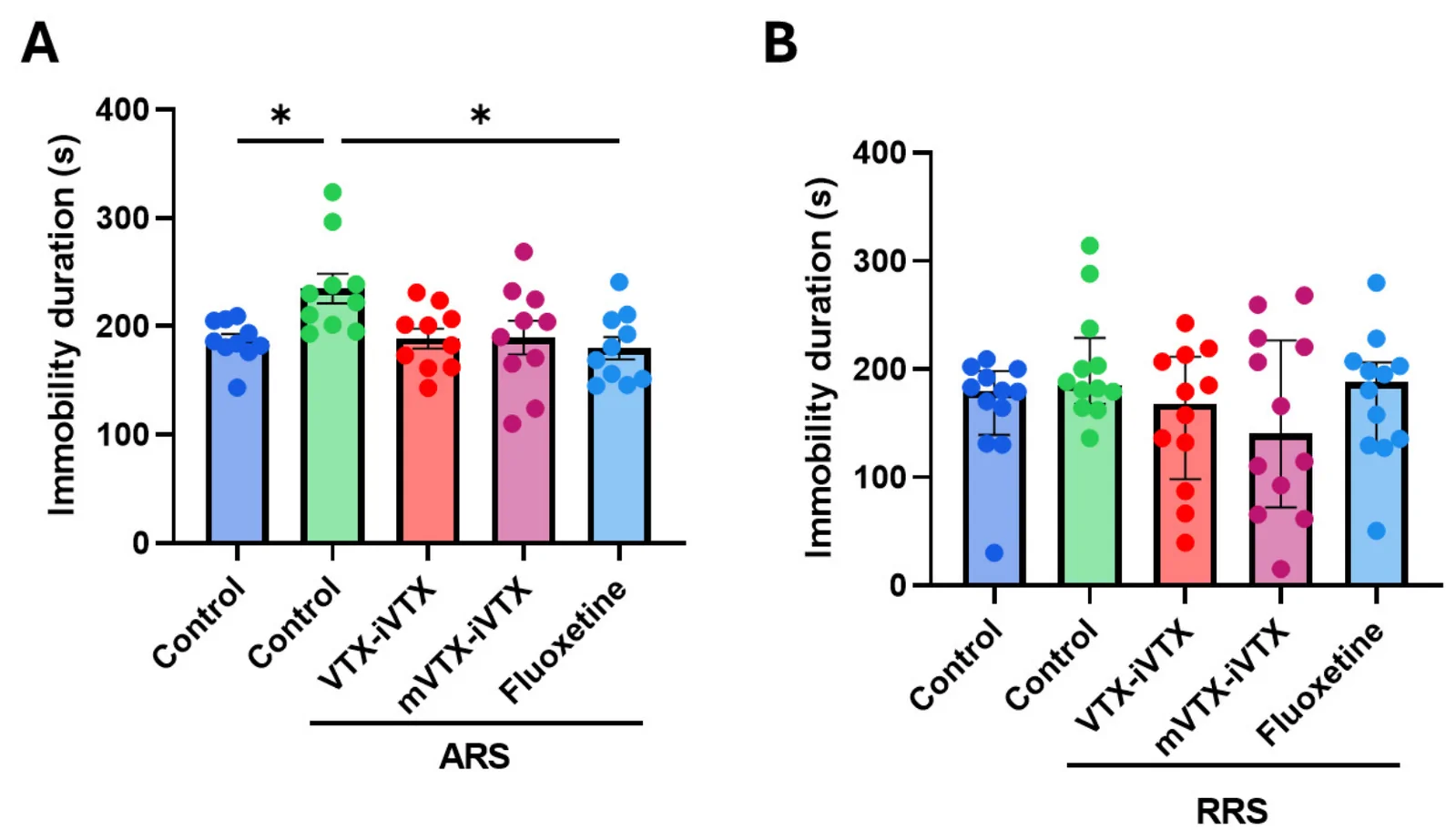
Immobility time (seconds) in the ARS (**A**) and RRS (**B**) model during the TST. Data are expressed as mean ± S.E.M. or median with interquartile range (IQR). ARS: *n* = 10 mice/group; RRS: *n* = 12 mice/group. Statistical analysis was performed using one-way ANOVA followed by Tukey’s post hoc test (**A**) or Kruskal–Wallis test followed by Dunn’s post hoc test (**B**). * *p* < 0.05. ARS, acute restraint stress; RRS, repeated restraint stress.

According to Figure 7A, the stress control group presented 1.26 times longer immobility time than that of the normal control group (*p* = 0.0315). Mice administered VTX-iVTX and mVTX-iVTX showed numerical reductions in immobility duration of approximately 1.24-fold compared with the stress control group; however, neither comparison reached statistical significance (*p* > 0.05). In contrast, the fluoxetine group showed a statistically significant 1.31-fold reduction in immobility time compared with the stress control group (*p* = 0.0113).

Figure 7B illustrates a more prolonged immobility duration in the stress control group compared to the normal control group, though this change was not statistically significant. Similarly, the administration of VTX-iVTX, mVTX-iVTX and fluoxetine exhibited no statistically significant differences in comparison with the stress control group, with decreases of approximately 1.31, 1.35 and 1.16 times, respectively. Furthermore, no statistically significant differences were recorded among these treated groups.

### 2.8. Molecular Docking on the Serotonin Transporter (SERT)

Vitexin (−9.19 kcal/mol) utilized its sugar appendage to form conventional hydrogen bonds with Phe335 (2.51 Å), Tyr95 (2.44 Å), and Ser438 (2.03 Å), plus a carbon hydrogen bond with Asp98 (3.68 Å). Its B ring engaged in π-anion interactions with Asp98 and Glu493, while the A ring formed a π-alkyl contact with Ile172.

**Figure 8:**
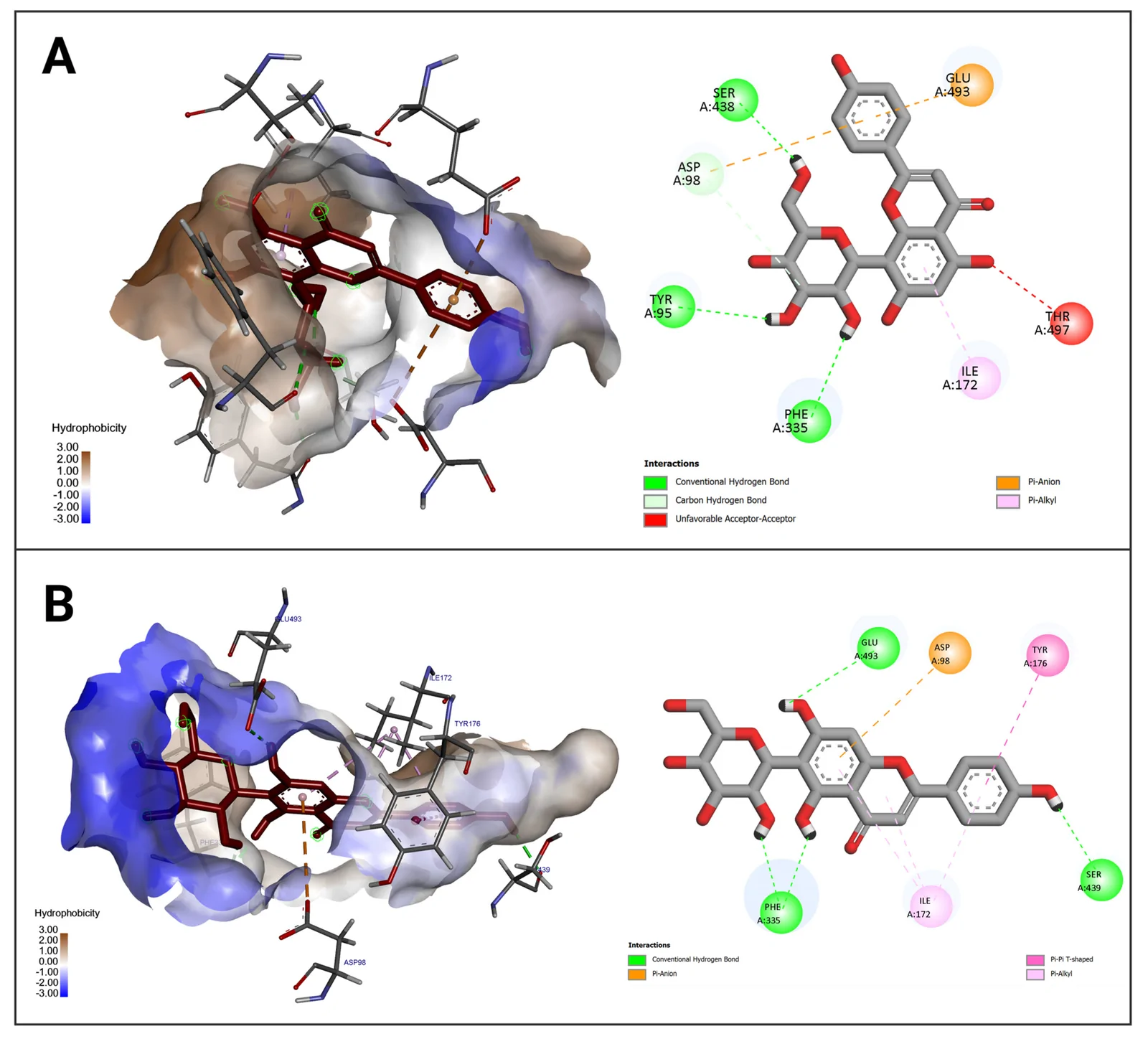
Molecular docking analysis with the human serotonin transporter. (**A**) Binding pose and 2D interaction diagram of vitexin. (**B**) Binding pose and 2D interaction diagram of isovitexin.

The C-6 isomer, isovitexin (−9.66 kcal/mol), established conventional hydrogen bonds with Phe335 (2.04 Å, sugar; 1.91 Å, A-ring), Ser439 (2.23 Å, B-ring), and Glu493 (2.93 Å, A-ring). Its A ring mediated a key π-anion interaction with Asp98, complemented by a hydrophobic network featuring a π-π T-shaped contact with Tyr176 (B-ring) and multiple π-alkyl interactions with Ile172 across all three rings (Figure 8).

### 2.9. Molecular Docking on the Serotonin 1A Receptor

Vitexin (−8.66 kcal/mol) effectively occupies the orthosteric cavity, stabilized by a coordinated hydrogen-bonding network. Its glucopyranoside moiety forms a conventional hydrogen bond with Ile189 (2.46 Å) and a carbon hydrogen bond with Thr188 (1.76 Å), while the B-ring hydroxyl interacts with Ser199 (2.16 Å, conventional H-bond). Additionally, the C-ring mediates a π-cation interaction with Lys191, complemented by π-alkyl hydrophobic contacts involving both the B and C rings with Ile189 and Ala365.

**Figure 9:**
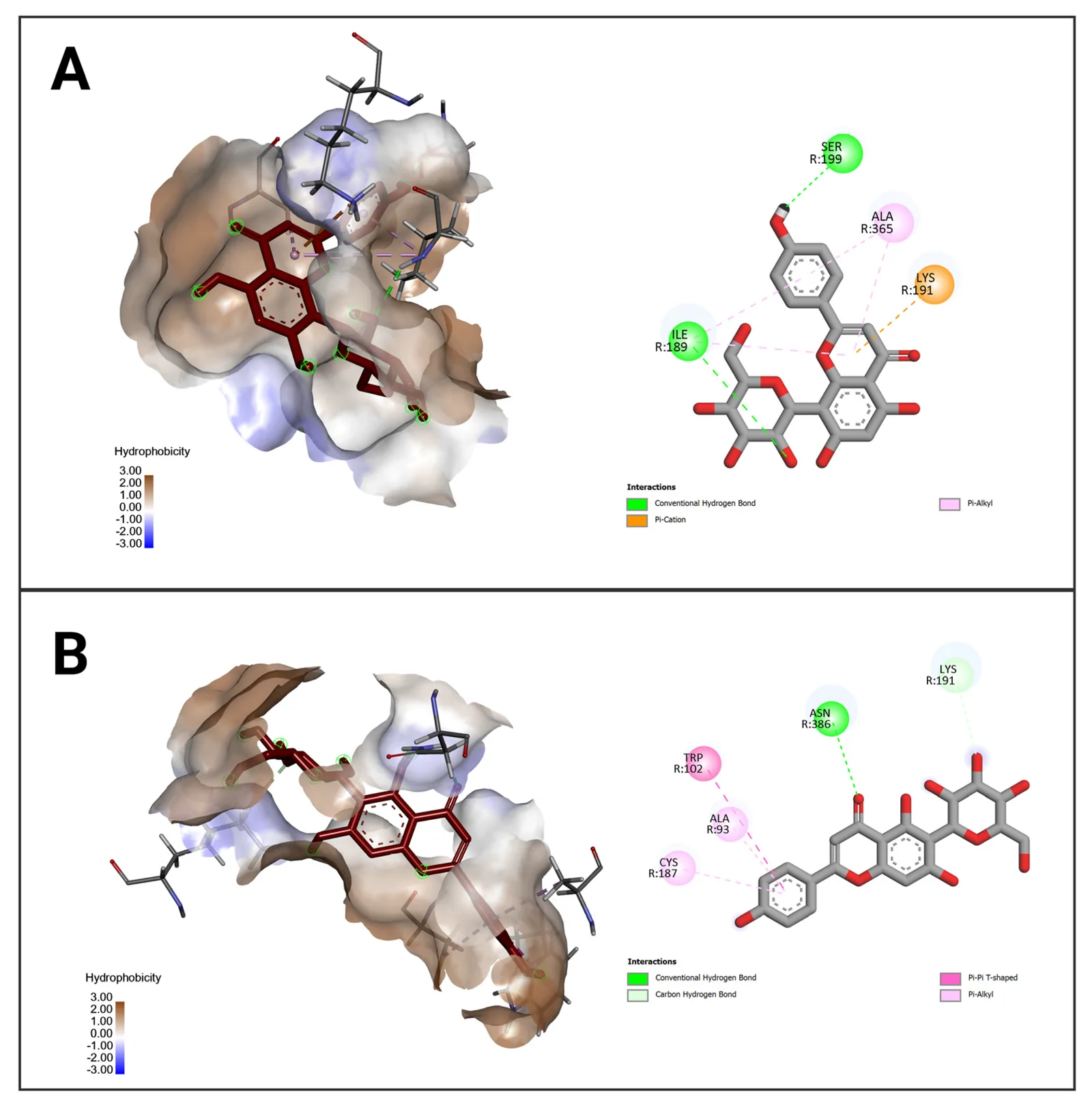
Molecular docking analysis with the serotonin 1A receptor. (**A**) Binding pose and 2D interaction diagram of vitexin. (**B**) Binding pose and 2D interaction diagram of isovitexin.

Isovitexin (−8.95 kcal/mol) benefits from its elongated framework, enabling the B-ring to penetrate deeper and optimally mimic befiradol’s native contacts. It establishes a conventional hydrogen bond with Asn386 (2.77 Å, via C-ring carbonyl) and a carbon hydrogen bond with Lys191 (2.73 Å, via sugar hydroxyl). This conformation is firmly secured by a profound hydrophobic network, featuring a π-π T-shaped interaction with Trp102, strong amide-π stacking with Met92 and Ala93, as well as multiple π-alkyl contacts with Ala93 and Cys187 (Figure 9).

### 2.10. Molecular Docking on the GABA_A_ Receptor

Vitexin (−10.43 kcal/mol) utilized its glucopyranoside moiety to form conventional hydrogen bonds with Tyr58 (2.55 Å) and His102 (2.43 Å), and carbon hydrogen bonds with Ser205 (2.85 Å) and Asn60 (3.32 Å). Its A-ring C5-OH established additional conventional hydrogen bonds with Ser159 (3.01 Å) and Tyr160 (2.28 Å), while the C-ring mediated a π-donor hydrogen bond with Ser205. This conformation was firmly anchored by π-π stacked interactions with Phe77 and Tyr210 (A and C rings), Tyr160 (C-ring), and a π-alkyl contact with Ala79 (B-ring).

**Figure 10:**
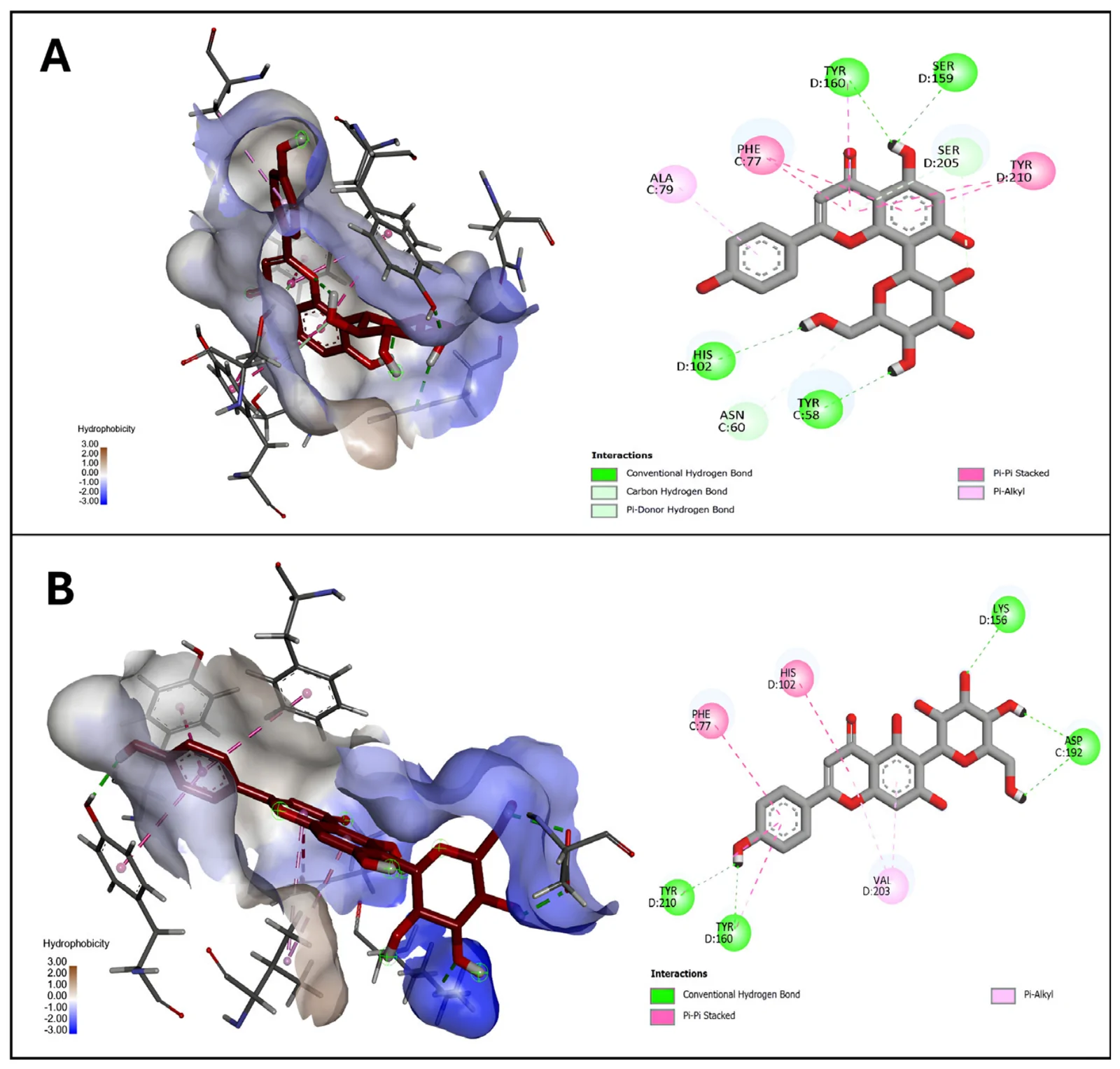
Molecular docking analysis with the GABA_A_ receptor. (**A**) Binding pose and 2D interaction diagram of vitexin. (**B**) Binding pose and 2D interaction diagram of isovitexin.

Isovitexin (−9.63 kcal/mol) engaged in a robust polar network via its glucopyranoside moiety, forming dual contacts with Lys156 (2.82 and 2.84 Å) and Asp192 (2.20 and 2.57 Å). Its B-ring C4′-OH formed critical conventional hydrogen bonds with Tyr160 (2.17 Å) and Tyr210 (2.64 Å). The dense hydrophobic scaffold featured π-π stacking between the B-ring and Phe77, Tyr160, and Tyr210, alongside a C-ring π-π stacking interaction with His102. Furthermore, Val203 formed π-alkyl contacts with both the A and C rings (Figure 10).

### 2.11. Molecular Docking on the Glucocorticoid Receptor

Within the expanded GR pocket, vitexin (−7.04 kcal/mol) utilized its glucopyranoside hydroxyls to form conventional hydrogen bonds with Arg611 (2.32 Å), Glu540 (2.62 Å), and Tyr663 (2.66 Å). Its A-ring C5-OH established an additional conventional hydrogen bond with Arg614 (2.54 Å). Further stabilization occurred via π-alkyl contacts with Val543 (B and C rings), and with Arg611 and Arg614 (A-ring).

**Figure 11:**
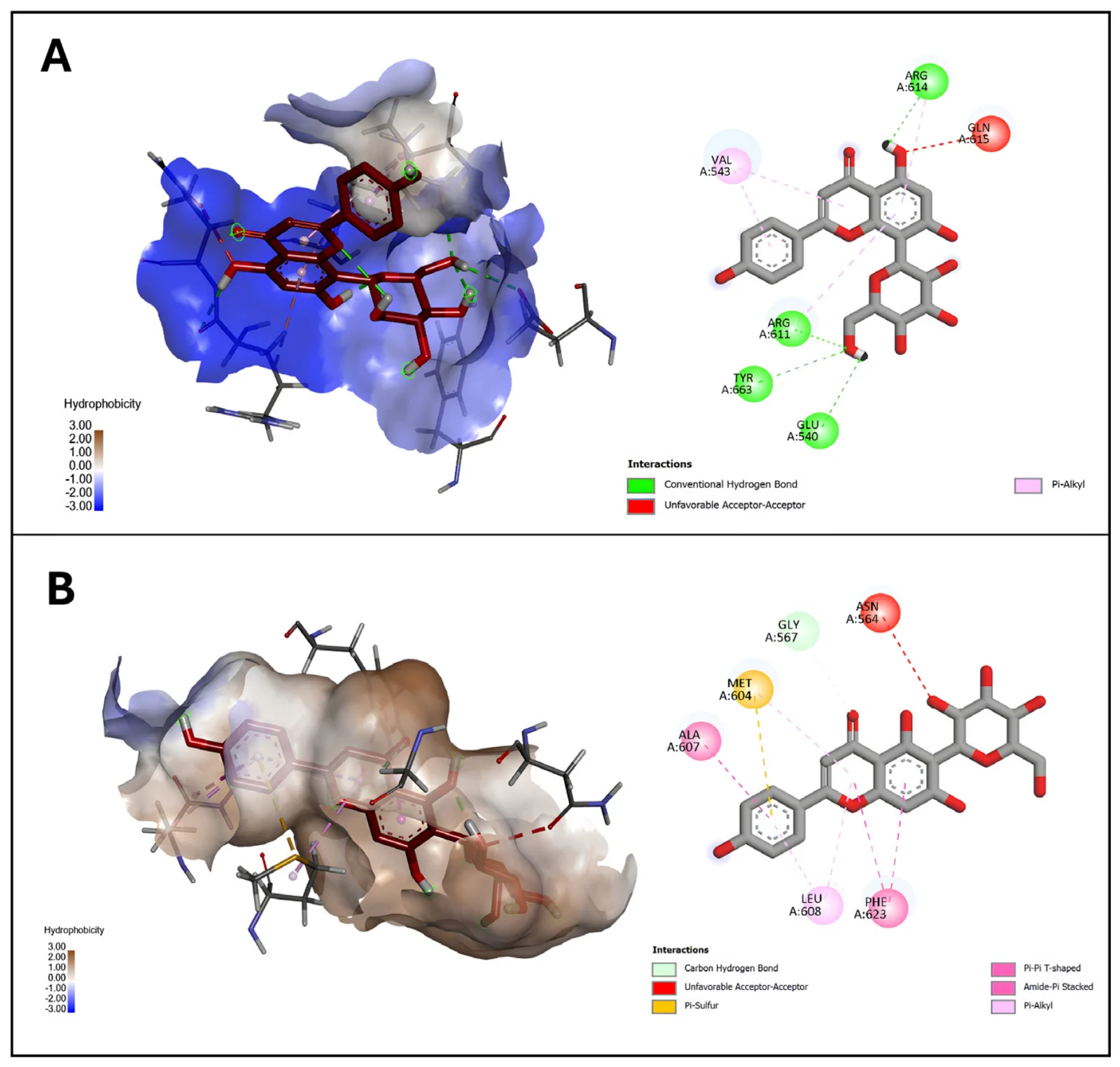
Molecular docking analysis with the glucocorticoid receptor. (**A**) Binding pose and 2D interaction diagram of vitexin. (**B**) Binding pose and 2D interaction diagram of isovitexin.

Isovitexin demonstrated a remarkably superior affinity (−10.16 kcal/mol), forming a carbon hydrogen bond with Gly567 (1.78 Å) via its C-4 carbonyl. Its deep accommodation was driven by an extensive hydrophobic network, featuring π-π T-shaped interactions with Phe623 (A and C rings), alongside multiple B-ring mediated contacts: critical amide-π stacking (Ala607 and Leu608), π-alkyl interactions (Met604, Met608, and Ala607), and a distinctive π-sulfur interaction with Met604 (Figure 11).

### 2.12. Molecular Docking on the Keap1 Domain

Vitexin (−7.64 kcal/mol) utilized its glucopyranoside hydroxyls as strong anchors to form conventional hydrogen bonds with Asn382 (3.04 Å, 2.77 Å) and Ser602 (3.09 Å). Its flavone core established critical conventional hydrogen bonds with Arg483 (2.11 Å, C-4 carbonyl) and Gly433 (2.76 Å, B-ring 4′-OH). This pose was fortified by strong electrostatic π-cation interactions with the crucial Arg415 (via A and C rings) and a π-alkyl contact with Ile461 (B-ring).

**Figure 12:**
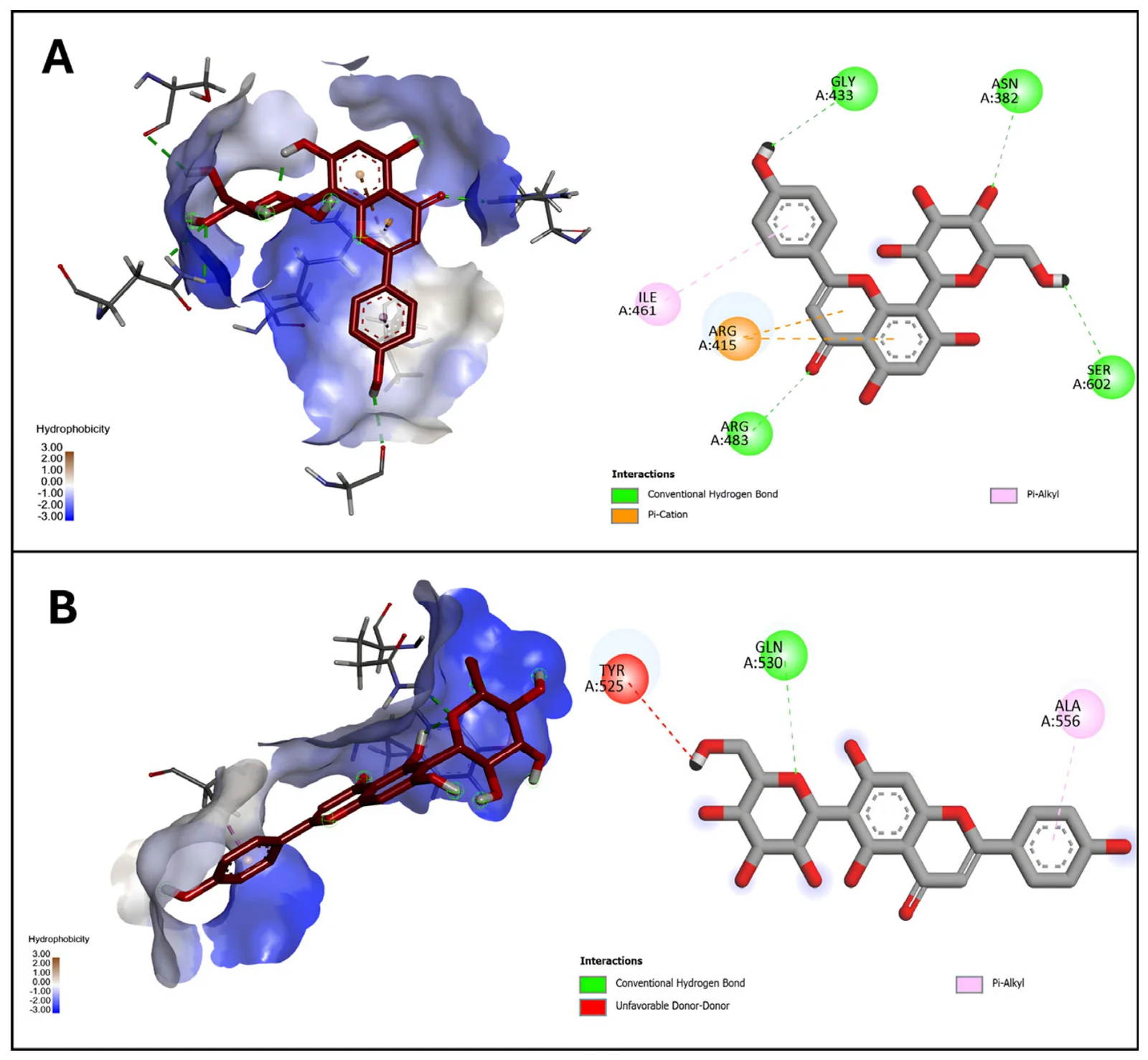
Molecular docking analysis on the Keap1 domain. (**A**) Binding pose and 2D interaction diagram of vitexin. (**B**) Binding pose and 2D interaction diagram of isovitexin.

Due to its streamlined C-6 geometry missing the critical Arginine triad (Arg415/Arg483), isovitexin showed a reduced affinity of −7.05 kcal/mol. Its molecular orientation was solely supported by a single conventional hydrogen bond with Gln530 (2.40 Å, sugar oxygen) and a π-alkyl hydrophobic interaction with Ala556 (phenyl B-ring) (Figure 12).

## 3. Discussion

Vitexin has been widely reported for its pharmacological activities, including antioxidant, anti-inflammatory [23], anti-Alzheimer’s disease [24] and blood pressure-lowering [25]. Previous studies have investigated vitexin as a single compound in various experimental stress paradigms, such as hypoxic–ischemic brain injury [26], chemically induced seizure models [27], and acute behavioral despair [10]. However, these studies mainly focused on vitexin while overlooking isovitexin, its structural isomer, which also coexists with vitexin in various plants. Thus, the potential effects of the fixed VTX-iVTX mixture remain insufficiently explored. To date, no studies have systematically evaluated the neurological effects of this fixed combination, particularly under stress conditions.

In this study, the DPPH assay was used as a preliminary assessment of the radical-scavenging activity of the VTX-iVTX mixture, with ascorbic acid as a positive control. VTX-iVTX exhibited an IC_50_ of 52.50 µg/mL, with approximately 13-fold lower radical-scavenging potency than ascorbic acid (Figure 1). Vitexin and isovitexin are C-glycoside apigenins that also show potential antioxidant activity. Chowjarean et al. reported that isovitexin showed scavenging activity with IC_50_ value of 1.72 mg/mL [28]. Similarly, vitexin has been reported to mark DPPH scavenging effects with 60% inhibition at 100 μg/mL [29]. Previous studies have also evaluated vitexin and isovitexin using both DPPH and ABTS assays, demonstrating that their radical-scavenging potency may vary depending on the assay system and experimental conditions [28,30]. The assay conditions in earlier reports differed markedly from ours, which limits the extent to which their IC_50_ values can be compared with our mixture in this study. The lower radical-scavenging activity of VTX-iVTX compared with luteolin and its C-glycoside derivatives may be attributed to the absence of a catechol moiety in the B-ring, which is important for radical stabilization after hydrogen donation [31]. Since antioxidant assays differ in radical species and reaction mechanisms, the present DPPH findings provides a preliminary indication of the radical-scavenging potential of VTX-iVTX rather than its overall antioxidant capacity.

Despite their antioxidant potential, vitexin and isovitexin have limited oral bioavailability due to their poor aqueous solubility, low intestinal permeability, and extensive phase II metabolism [32]. These biopharmaceutical limitations may restrict the therapeutic potential of vitexin and isovitexin, prompting the development of delivery systems to improve solubility, stability, and absorption. Strategies including micronized powders, nanosuspensions, liposomes, nanoemulsions, and coated nanoparticles have been investigated to enhance their biopharmaceutical properties [12,13,33,34,35]. Microencapsulation provides a protective matrix while enabling sustained drug release. Our previous study demonstrated sustained release of VTX-iVTX for up to 24 h under simulated gastrointestinal conditions, together with improved formulation stability and biopharmaceutical properties [11].

To evaluate the in vivo efficacy of the microencapsulated formulation, the restraint stress model has been employed to further investigate the stress-reducing effects of these two flavones. This model simulates behavioral and neuroendocrine alterations associated with stress-induced depressive disorders [36,37], thus supporting the utility of this model in reproducing fundamental features of human stress and anxiety. Furthermore, it allows precise control over stress duration and intensity, while other models, such as the SDS model, strongly depends on the aggressor animal [18] and the CUMS paradigm shows substantial variability across different laboratories [38]. Chronic restraint influences modulating neuroendocrine pathways and monoaminergic neurotransmission, which are correlated to depression and anxiety behaviors [39,40]. In rodent models, restraint stress can be established for applying both single acute and repeated stress induction paradigms [41,42]. This experimental flexibility and standardization make the model suitable for evaluating the efficacy of vitexin and isovitexin across various stress conditions.

Both ARS and RRS induction in our study resulted in an increase in MDA levels (Figure 2), indicating a tendency toward lipid peroxidation and oxidative membrane damage in the examined brain regions [43,44]. Treatment with both formulations was associated with lower MDA levels than the stress controls, although these differences did not reach statistical significance. This pattern is consistent with previous established evidence showing that vitexin and isovitexin lower MDA concentrations in various disease models [23,45,46]. The numerical MDA reductions were in the same direction as the radical-scavenging activity observed in the DPPH assay, although the non-significant MDA findings do not establish an in vivo antioxidant effect. Under acute stress, the microencapsulated form showed a slightly greater numerical reduction in MDA levels than the crude form in both the hippocampus (HPC) and hypothalamus (HYP). This observation is consistent with our previous pharmacokinetic study showing that microencapsulation markedly increased the Cmax and AUC of vitexin and isovitexin compared with the raw extract [11], although a direct relationship between systemic exposure and MDA reduction was not assessed in the present study. Under repeated stress, only fluoxetine achieved a significant MDA reduction restricted to the prefrontal cortex, while other groups showed non-significant declines. Interestingly, the crude form showed a numerically greater reduction across brain regions during repeated stress. Whether the single-dose pharmacokinetic advantage of microencapsulation is sustained after repeated administration remains unclear. Alterations in systemic exposure or metabolism during repeated administration may contribute to this discrepancy and warrant further investigation.

In contrast, GSH serves as a co-substrate for glutathione peroxidase, protecting cells from oxidative damage [47]. Under acute stress, the stress-control group showed lower GSH levels, with a statistically significant reduction relative to the normal-control group only in the hypothalamus (Figure 3B). Both VTX-iVTX and mVTX-iVTX significantly increased hypothalamic GSH compared with the stress-control group. Under repeated stress, GSH levels in the stress-control group were numerically lower than those in the normal-control group across the three brain regions, but none of these differences reached statistical significance (Figure 3C–E). mVTX-iVTX significantly increased GSH relative to the stress-control group in the hippocampus, hypothalamus, and prefrontal cortex, whereas VTX-iVTX produced a significant increase only in the hypothalamus. These treatment-associated increases should not be interpreted as normalization because the RRS-control group did not differ significantly from the normal-control group. Previous evidence indicates that vitexin can influence antioxidant defenses by improving enzyme activities [48], the GSH/GSSG ratio [49] and GSH levels in several toxicity models [50,51]. Nrf2-related pathways have also been implicated in vitexin-associated antioxidant responses [52,53,54], although their involvement in the GSH changes observed in our study remains to be established.

In the repeated-stress experiment, the two formulations showed different patterns across oxidative stress markers: VTX-iVTX was associated with a greater numerical reduction in MDA, whereas mVTX-iVTX produced significant increases in GSH relative to the stress-control group across all three brain regions. These findings should be interpreted as treatment-associated changes rather than restoration or normalization of oxidative status, particularly because the RRS-control group did not differ significantly from the normal-control group for GSH. GSH is an endogenous antioxidant involved in peroxide detoxification, whereas MDA is a downstream product of lipid peroxidation and reflects accumulated oxidative damage. Accordingly, changes in GSH and MDA need not occur in parallel at a single time point because lipid peroxidation is also influenced by ROS generation and other enzymatic and non-enzymatic defense systems [55,56]. In the hippocampus, GSH levels in the mVTX-iVTX group were significantly higher than those in both the VTX-iVTX and fluoxetine groups; however, the biological significance of this between-treatment difference requires further investigation [57].

Given the role of the HYP in the HPA axis, serum cortisol was measured as an exploratory glucocorticoid-related marker of the stress response. Although corticosterone is the predominant glucocorticoid in rodents, cortisol has also been measured in rodent studies and has been reported to change under stress-related conditions [58,59,60]. However, because the VIDAS Cortisol S assay was not specifically validated for mouse serum, these measurements should be interpreted only as relative changes in serum cortisol among experimental groups and cannot support strong claims about HPA-axis modulation.

The stress control group displayed an increase in serum cortisol levels (Figure 4), which was particularly pronounced in acute 4-h stress-induced mice (Figure 4A). This increase is consistent with the rapid glucocorticoid response reported during acute stress exposure [61]. Under ARS, cortisol levels were lower in all treatment groups than in the stress control group, but only mVTX-iVTX and fluoxetine produced statistically significant reductions. The greater numerical decrease observed with mVTX-iVTX than with VTX-iVTX may be related to the higher C_max_ and prolonged plasma exposure previously reported for the microencapsulated formulation [11]. Given the 30-min pretreatment period, this may have contributed to the more pronounced cortisol reduction during rapid HPA-axis activation, although this was not directly examined in the present study. In contrast, under RRS, neither the difference between the normal and stress control groups nor the decreases observed in the treatment groups reached statistical significance (Figure 4B). Therefore, the RRS cortisol findings should be interpreted as descriptive trends rather than evidence of HPA-axis normalization. This pattern may reflect HPA-axis habituation during repeated stress [62,63], which could contribute to the less distinct treatment effects observed under RRS. Fluoxetine influences HPA axis activity via serotonergic pathway [64,65], whereas vitexin has been reported to influence stress-related responses through serotonergic (5-HT_1A_), noradrenergic (α2), and dopaminergic (D1–D3) receptors. In our docking analysis, VTX and iVTX showed favorable predicted interactions with GR and SERT, providing a possible basis for further investigation of glucocorticoid and serotonergic pathways [66,67]. However, these docking findings are predictive and require functional validation.

The behavioral and biochemical endpoints changed in parallel in several treatment groups; however, no correlation analysis was performed. Therefore, a direct relationship between these changes cannot be established. The behavioral findings are discussed below in relation to the known functions of the corresponding brain regions.

The HPC is critically involved in learning and memory, while the PFC also contributes to recognition memory and working memory processes [68,69]. Accordingly, the changes observed in these regions were considered alongside NOR performance. Under ARS, both VTX-iVTX and mVTX-iVTX showed higher novel-object exploration than the stress control, although these differences did not reach statistical significance. In contrast, both formulations significantly increased exploration of the novel object under RRS (Figure 5). Because the NOR ratio was not compared with the theoretical 50% chance level, these findings should be interpreted as relative novel-object exploration rather than definitive evidence of restored recognition memory. Previous studies have reported effects of vitexin and isovitexin in learning- and memory-related paradigms [70,71,72]. Serotonergic signaling through 5-HT1A receptors has been implicated in recognition memory [73,74] and may be relevant to the behavioral changes observed in our study. Despite its pharmacokinetic advantage, mVTX-iVTX did not consistently outperform VTX-iVTX in the NOR test. Given the absence of comparison with the 50% chance level, this between-formulation difference should not be interpreted as evidence of differential memory efficacy.

Social behavior is closely associated with PFC function, particularly the medial PFC, which regulates social approach and is vulnerable to stress-induced dysfunction [75]. Consistent with this, under ARS, both formulations significantly increased social interaction and reduced the corner ratio, with a more pronounced effect of mVTX-iVTX (Figure 6A,B). These findings are also in line with the previously reported anxiolytic effects of vitexin [27]. In particular, mVTX-iVTX showed a more pronounced improvement in social interaction than VTX-iVTX, which may partly reflect the pharmacokinetic advantages of the microencapsulated formulation, including improved dissolution and systemic availability, a higher C_max_, and sustained plasma concentrations during the early phase of acute stress [11]. The elevated corner ratio in the ARS group was also consistent with the greater cortisol response and acute HPA-axis activation, which may promote avoidance and reduce social engagement [76]. Under RRS, both formulations showed higher social interaction ratios and lower corner ratios than the stress control, although these differences did not reach statistical significance (Figure 6C,D). Representative movement trajectories further showed greater exploration of the social zone following treatment (Figure 6E), although this pattern was not reflected by significant changes in the quantitative measures. Serotonergic and GABAergic pathways, which are involved in social and anxiety-related behaviors, may also contribute to these behavioral patterns [77,78].

The TST reflects stress-related passive coping behavior and is influenced by HPA-axis activity and oxidative stress [79,80]. In the present study, VTX-iVTX and mVTX-iVTX showed numerical reductions in immobility under ARS, although these changes were not statistically significant, while no treatment significantly altered immobility under RRS (Figure 7). Changes in hypothalamic redox markers and cortisol may be related to the behavioral pattern observed in the TST, while changes in the HPC and PFC may also be relevant to adaptive behavioral responses through HPC–PFC signaling [81,82]. The longer immobility observed after acute restraint may reflect a stronger short-term stress response, whereas repeated exposure may involve adaptive changes in HPA-axis regulation and coping responses [83]. These findings should also be interpreted in the context of the testing sequence, as the TST was performed after restraint and the preceding behavioral procedures.

Earlier research demonstrated that vitexin reduced immobility duration in mice, with its antidepressant-like mechanism involving enhanced catecholamine concentrations and interaction with 5-HT_1A_, noradrenergic α2, and dopaminergic D1, D2, and D3 receptors [10]. Meanwhile, other flavonoids may additionally modulate depressive-like behavior through oxidative stress, neuroinflammation, neuroplasticity, and HPA-axis regulation [84,85], suggesting that similar additional pathways may also contribute to the antidepressant-like effects of vitexin and isovitexin. These potential mechanisms were further explored through molecular docking.

To explore the potential molecular mechanisms underlying the effects of vitexin and isovitexin under stress, we first investigated their interaction with the serotonin transporter (SERT). The SERT is a fundamental physiological target responsible for terminating serotonergic signaling via the reuptake of neurotransmitters into presynaptic neurons [86,87]. The X-ray structure of human SERT complexed with paroxetine was selected for its outward-open conformation, which is suitable for investigating competitive inhibition at the central binding site. Docking simulations were then performed to compare the predicted binding profiles of the investigated flavonoids with fluoxetine, the SSRI used as a positive control in the in vivo restraint stress models. Utilizing this specific holo-structure allows for a more detailed assessment of whether the bulky C-glycoside flavone scaffolds could potentially access and interact with the essential Asp98 residue, which has been implicated as an important structural feature in SSRI binding profiles.

The predicted interacting residues and corresponding interaction types of VTX and iVTX across the selected targets are summarized in Supplementary Table S1. A comparative structural analysis of the binding modes reveals distinct pharmacophoric strategies employed by the synthetic SSRI paroxetine and the natural C-glycosyl flavones within the SERT central cavity. Structurally, paroxetine is characterized by flexible ring systems, including a piperidine and a 1,3-benzodioxole ring, which facilitate optimal hydrophobic packing through π-π T-shaped and amide-π stacked interactions. Most importantly, paroxetine possesses a highly basic piperidine nitrogen atom, which serves as an indispensable anchor by forming a robust salt bridge and strong electrostatic interactions with the critical Asp98 and Ser336 residues. In contrast, vitexin and isovitexin lack the basic nitrogen of classical SSRIs and may instead use their rigid, electron-rich flavonoid scaffolds to form alternative interactions with Asp98. Additionally, the differing positions of their bulky glucopyranosyl moieties distinctly influence their steric accommodation within the binding pocket. Isovitexin, with a C-6 glycosidic linkage, showed a more favorable predicted binding score than C-8-linked vitexin, likely due to its more elongated conformation that minimizes steric clashes within the narrow SERT pocket. This streamlined geometry enables deeper cavity penetration and stronger hydrophobic contacts, including π-π T-shaped interactions with Tyr176 and π-alkyl contacts with Ile172. The C-8 linkage of vitexin may result in a more perpendicular orientation and different spatial accommodation within the binding cavity. Docked into the outward-facing, paroxetine-bound human SERT structure, both flavonoids were predicted to occupy the central binding site in a manner overlapping with the paroxetine binding region.

In addition to SERT, the serotonin 5-HT1A receptor was included as an exploratory docking target because of its established involvement in anxiety- and depression-related neuropharmacology. Previous behavioral work has implicated 5-HT1A-related pathways in the antidepressant-like effects of vitexin [10]. Whether isovitexin shares this mechanism remains unknown. For the molecular docking simulations, the high-resolution cryo-EM structure of the human 5-HT1A receptor in complex with Gi protein and befiradol was selected to represent an active receptor conformation associated with G-protein coupling. Befiradol stabilizes an expanded orthosteric pocket, providing a structural template for assessing whether the bulky glucosyl moieties of vitexin and isovitexin can be accommodated within this cavity. These docking simulations assess predicted structural compatibility and do not establish receptor agonism or antidepressant activity.

Befiradol is characterized by an elongated, highly flexible structure that, while anchored by a critical salt bridge with Asp116, is predominantly stabilized by an extensive and massive hydrophobic network. Vitexin and isovitexin share a planar tricyclic flavone core bearing a bulky glucopyranosyl moiety. The position of the sugar may influence their spatial accommodation, with the C-6 linkage of isovitexin producing a more elongated geometry than the C-8 linkage of vitexin. This favorable orientation allows B-ring (phenyl ring) of isovitexin to insert deeper into the cavity, reproducing key hydrophobic contacts of befiradol, including amide-π stacked interactions with Met92 and Ala93 alongside π-alkyl contacts with Cys187. The interpretation of the docking results for the 5-HT1A receptor should consider the conformational state of the template, which is a cryo-EM structure in complex with Gi protein and befiradol. Because the structural model represents an active receptor form bound to Gi protein and befiradol, the favorable fit of both flavone glycosides primarily reflects geometric compatibility with this agonist stabilized cavity, which should not be interpreted as conclusive evidence of functional agonism.

In addition to the serotonergic system, the anxiolytic-like efficacy of these isomers likely involves the modulation of inhibitory neurotransmission. The type-A γ-aminobutyric acid (GABA_A_) receptor is a crucial ligand-gated chloride channel whose allosteric modulation is fundamentally linked to anxiolytic and sedative pharmacological effects. For the molecular docking simulations, the high-resolution cryo-EM structure of the human full-length heteromeric α1β3γ2L GABA_A_ receptor in complex with alprazolam and GABA was selected. This potentiated conformation provides a well-resolved template of the classical benzodiazepine site at the α1/γ2 interface, enabling an in silico evaluation of whether vitexin and isovitexin might engage this allosteric pocket [88].

A comparative structural analysis within the classical benzodiazepine allosteric binding pocket at the α1/γ2 interface of the GABA_A_ receptor reveals differing theoretical interaction profiles between the synthetic positive allosteric modulator, alprazolam, and the natural C-glycosyl flavones. Alprazolam predominantly relies on the nitrogen atoms within its diazepine and triazole rings to form critical hydrogen bonds (such as with Ser205), and utilizes a distinctive chlorine atom to establish halogen interactions with His102, complemented by a robust hydrophobic network. Vitexin and isovitexin lack halogen and basic nitrogen atoms but may compensate through their rigid, planar flavone cores, which form predicted π-π stacking interactions with aromatic residues such as Phe77, Tyr160, and Tyr210. Furthermore, multiple hydroxyl groups on both the flavone core and the glucosyl moiety serve as donors and acceptors, forming extensive hydrogen bonds with peripheral residues including Tyr58, His102, Lys156, and Asp192. Unlike O-glycosides, C-glycosidic bonds are generally more resistant to acidic and enzymatic hydrolysis, which may contribute to the stability of these flavonoids. However, whether the sugar moieties remain intact and contribute to receptor binding in vivo requires experimental validation. Interestingly, the position of the sugar moiety may influence the spatial accommodation of the two isomers depending on the receptor architecture. Whereas the C-6 linkage of isovitexin was more favorable in the elongated orthosteric channel of 5-HT_1A_, docking at GABA_A_ showed a more favorable predicted binding score for C-8 glycosidic linkage vitexin. Vitexin exhibited higher predicted affinity than isovitexin, as its perpendicular C-8 arrangement conforms favorably to the α1/γ2 cavity. This geometry allows the sugar hydroxyls of vitexin to contact Tyr58 and His102 while orienting the flavone core to engage in favorable π-π stacking and π-donor interactions. It should be noted that the target structure (PDB ID: 6HUO), resolved by cryo-EM, captures the GABA_A_ receptor bound to alprazolam, stabilizing the classical benzodiazepine allosteric pocket in an open, active conformation. Thus, the favorable docking scores for vitexin and isovitexin primarily reflect their predicted geometric and electrostatic compatibility with this preorganized receptor conformation and should not be interpreted as evidence of receptor activation.

Beyond the neurotransmitter modulation, the HPA axis plays a pivotal role in mediating the systemic responses to stress. Specifically, the glucocorticoid plays an integral role in negative feedback regulation of the HPA axis, contributing to the modulation of circulating stress hormones such as cortisol. Because severe restraint stress often induces glucocorticoid resistance and elevated cortisol levels, evaluating putative interactions with the glucocorticoid receptor provides a structural perspective on potential neuroendocrine regulation by these flavonoids [21]. The crystal structure of the human glucocorticoid receptor ligand binding domain complexed with deacylcortivazol was selected for molecular docking. The binding of deacylcortivazol is associated with an expanded binding cavity of approximately 1070 Å^3^, which is substantially larger than the classical dexamethasone binding pocket. This expanded conformation provides an accommodating structural environment that theoretically facilitates the placement of bulky flavone glycosides like vitexin and isovitexin while minimizing steric clashes.

Isovitexin exhibited higher predicted binding affinity than vitexin, likely facilitated by the spatial orientation of its C-6 glucosyl linkage. This elongated geometry allows isovitexin to insert deeper into the expanded cavity stabilized by deacylcortivazol, whereas the more perpendicular C-8 arrangement of vitexin limits insertion to shallower regions with fewer hydrophobic contacts. The predicted pose of isovitexin also showed interactions between its B-ring and Ala607, Leu608, and Met604, including amide-π stacked and π-sulfur interactions. These interactions resemble contacts observed for the co crystallized ligand deacylcortivazol and may contribute to the favorable docking score of isovitexin. These favorable scores primarily reflect geometric complementarity to this specific agonist expanded pocket rather than intrinsic hormonal activity.

Keap1 is an intracellular regulator of the Nrf2 pathway and contains a Kelch-domain binding pocket that has been structurally characterized with small-molecule ligands [89]. Because oxidative stress-related endpoints were included in the present study, the Keap1 Kelch domain was selected as an exploratory docking target to assess the predicted structural compatibility of vitexin and isovitexin with this pocket. For the docking simulations, the human Keap1 Kelch domain bound to the small-molecule fragment 4-oxo-4H-1-benzopyran-2-carboxylic acid was selected. The co-crystallized ligand contains a chromone scaffold that is structurally related to the flavone backbone of the investigated compounds. The docking results therefore provide a structural hypothesis only and do not demonstrate Keap1 inhibition, Nrf2 activation, or neuroprotective antioxidant activity.

Comparative analysis of the Keap1 binding pocket revealed distinct predicted interaction patterns for the two isomers. The native ligand, which contains a chromone scaffold similar to the flavone core, interacts with key residues including Arg415 and Arg483 through electrostatic and hydrogen bond interactions. Vitexin approximates this pattern through its perpendicular C-8 geometry, allowing the C-4 carbonyl oxygen to form a hydrogen bond with Arg483 alongside strong π-cation electrostatic with Arg415. Conversely, the elongated C-6 conformation of isovitexin appears less compatible with the pocket topography, limiting interactions with these key arginine residues. Docking was performed using the Kelch domain structure of Keap1 bound to a small molecule inhibitor fragment. The predicted interactions of vitexin and isovitexin with residues in the Nrf2 binding pocket may indicate potential compatibility with this site, although the static docking model primarily assesses spatial occupancy and does not establish disruption of the dynamic Keap1 Nrf2 protein interaction.

The computational results suggest that the positional isomerism of the glycosidic linkage influences the predicted spatial accommodation and interaction patterns of these flavonoids across the selected targets. The C-6 geometry of isovitexin was associated with favorable predicted accommodation in the glucocorticoid receptor, SERT, and 5-HT1A receptor models, whereas the C-8 geometry of vitexin showed favorable predicted interactions within the GABAA benzodiazepine site and the Keap1 Kelch-domain pocket. These observations describe target-dependent structural compatibility in static docking models. They should not be interpreted as evidence that either compound produces multitarget anxiolytic, antidepressant, neuroprotective, receptor-activating, or receptor-inhibiting effects in vivo.

Importantly, several methodological and biological considerations regarding these in silico findings should be emphasized. In the present study, molecular docking served strictly as an exploratory, hypothesis generating tool to evaluate whether vitexin and isovitexin theoretically possess spatial compatibility with central stress regulatory targets, thereby providing the foundational rationale to engineer a microencapsulation platform capable of protecting these labile flavonoids for in vivo delivery. However, these static calculations model isolated ligand protein interactions and do not capture formulation properties, release kinetics, or matrix effects imparted by the microcapsules themselves. Furthermore, because vitexin and isovitexin were administered as a dual isomer mixture, their close structural homology raises the distinct possibility of competitive binding or mutual displacement at shared orthosteric or allosteric cavities in vivo. While individual binding simulations indicate divergent geometric preferences across narrow and wide pockets, validating whether these isomers exert distinct downstream mechanisms, competitive antagonism, or synergistic modulation will require dedicated competitive radioligand binding experiments and cellular functional assays in subsequent studies.

To the best of our knowledge, this study is among the first to comprehensively evaluate the combined effects of vitexin and isovitexin in models of restraint stress. The present findings indicate beneficial behavioral and redox-related effects of VTX-iVTX, although the magnitude and significance of these effects differed between acute and repeated restraint stress. Notably, the effects of VTX-iVTX on serum cortisol have not previously been investigated in these models. Nevertheless, serum cortisol was assessed as an exploratory glucocorticoid-related measure, and these findings should not be interpreted as evidence of HPA-axis modulation.

Despite the observed effects of vitexin and isovitexin on behavioral outcomes and stress-related biomarkers, several limitations of the present study should be acknowledged. Although the behavioral and biochemical endpoints changed in parallel in several treatment groups, no within-animal correlation or mediation analysis was performed. Therefore, the present data do not establish a direct or causal relationship between changes in MDA, GSH, or cortisol and the observed behavioral outcomes. First, only male Swiss albino mice were included, which limits the generalizability of the findings across sexes and mouse strains. Second, although the group sizes used in the ARS (n = 10 per group) and RRS (n = 12 per group) experiments provided adequate sensitivity for relatively large omnibus effects, the study may have been underpowered to detect small-to-moderate effects or subtle differences between treatment groups. Accordingly, non-significant comparisons, particularly those between the crude and microencapsulated formulations, should be interpreted cautiously. Third, only a single dose of VTX-iVTX (30 mg/kg) was evaluated; therefore, dose–response relationships and the minimum effective dose could not be established. In addition, the repeated restraint paradigm was limited to seven days, and longer-term efficacy and safety remain to be determined. Fourth, behavioral tests were performed sequentially, following restraint exposure and prior to blood and brain collection. The behavioral tests were ordered from less stressful to more stressful procedures, with TST performed last to minimize the potential influence of test-related stress on subsequent behavioral outcomes [90]. Our previous study using a comparable testing protocol suggested that behavioral testing alone did not significantly alter cortisol levels in non-stressed mice [91]. Accordingly, an additional control group was not included to avoid unnecessary animal use, in accordance with the Reduction principle of the 3Rs. Nevertheless, potential test-order or carry-over effects cannot be excluded, and the behavioral battery may have contributed to the subsequent MDA, GSH, and cortisol measurements. Thus, these measurements cannot be completely attributed to restraint stress. In addition, the NOR ratio was not formally tested against the theoretical 50% chance level; therefore, this endpoint should be interpreted as relative novel object exploration rather than definitive evidence of restored recognition memory. No independent control assay was performed, limiting the interpretation of TST immobility. Further locomotor-control assay can be conducted to measure anxiety-like behavior. Fifth, serum cortisol was assessed as an exploratory glucocorticoid-related measure of the stress response. Although corticosterone is the predominant glucocorticoid in rodents, cortisol can also be detected and has been associated with corticosterone under various stress-related conditions [92,93]. Nevertheless, corticosterone was not simultaneously measured and the VIDAS Cortisol S assay was not specifically validated for mouse serum. Therefore, the cortisol findings should be regarded as exploratory and should not be interpreted as evidence of HPA axis modulation. Sixth, the DPPH assay was the only in vitro antioxidant assay in our study, which limits a comprehensive assessment of the antioxidant capacity of VTX-iVTX combination. Complementary assays such as ABTS, ORAC, or FRAP should be included in further studies. Seventh, the present study demonstrates behavioral and biochemical effects but does not establish the underlying mechanisms. The proposed mechanisms are primarily based on molecular docking and previous evidence. Docking predicts structural compatibility and potential binding but does not establish direct target engagement or downstream signaling in vivo. Further studies using Western blotting, qPCR, immunofluorescence, functional receptor assays, and neurotransmitter analysis are needed to clarify the underlying mechanisms. Eighth, a blank alginate–chitosan microcapsule control was not included, limiting the assessment of potential formulation-related effects. The pharmacokinetic advantage of the microencapsulated formulation was inferred partly from our previous pharmacokinetic study rather than directly characterized in the mice used in the present experiments. Consequently, simultaneous pharmacokinetic–pharmacodynamic studies in mice are warranted to establish the relationship between systemic exposure and therapeutic efficacy. Brain and cerebrospinal fluid concentrations were also not measured, limiting the assessment of CNS exposure. Future studies should integrate plasma and brain pharmacokinetics with pharmacodynamic endpoints. Finally, the restraint stress paradigm represents a highly standardized, predominantly physical stressor and does not fully reproduce the psychological and social complexity of human stress-related disorders. Confirmation in complementary models, including CUMS and SDS, as well as subsequent translational investigations, will therefore be necessary before clinical relevance can be established.

## 4. Materials and Methods

### 4.1. Vitexin–Isovitexin-Loaded Microcapsules

The vitexin and isovitexin mixture (1:1 ratio), extracted from muSng bean seed coats (*Vigna radiata* L.), was purchased from Napro (Hanoi, Vietnam). The contents of vitexin and isovitexin were quantified and verified (purity > 95%) at the Department of Pharmaceutical Technology, School of Pharmacy, University of Medicine and Pharmacy at Ho Chi Minh City, via high-performance liquid chromatography (HPLC) [11]. The 1:1 vitexin–isovitexin ratio, as obtained following the original extraction from mung bean seed coat, was retained from the standardized fixed-ratio mixture used in our previous formulation and pharmacokinetic study [11]. The present study was not designed to establish an optimal VTX-iVTX ratio.

The vitexin–isovitexin-loaded microcapsules were prepared via a two-stage process: alginate core synthesis followed by chitosan coating as outer layers, as previously described in our research [11]. First, alginate cores were fabricated through an emulsification stage using the water-in-oil (W/O) technique. Vitexin–isovitexin mixture (200 mg) was dispersed into 1.17% (*w*/*v*) low-viscosity sodium alginate solution and mixed to obtain a homogenizer suspension. This mixture was introduced dropwise into 40 g of isooctane containing 2 g of Span 80 and homogenized using a T25 digital ULTRA-TURRAX^®^ homogenizer (IKA-Werke GmbH & Co. KG, Staufen, Germany). to form a W/O emulsion. Subsequently, a 5.78% (*w*/*w*) Tween 80 solution was added, followed by a 7.60% (*w*/*w*) calcium chloride solution under continuous homogenization at 7200 rpm. The resulting alginate cores were collected by centrifugation and filtration, and subsequently dried at room temperature. In the second stage, the alginate cores were immersed in a 0.5% (*w*/*v*) chitosan solution under magnetic stirring at 100 rpm for 2 h. The chitosan-coated alginate microcapsules were recovered by centrifugation, filtered, and stored in a desiccated, sealed container for further investigation.

The spherical microcapsules exhibited a mean diameter of 10.78 µm, with a drug loading of 22.45% and an encapsulation efficiency of 68.92%. Differential scanning calorimetry revealed an endothermic melting peak at 257.08 °C with an enthalpy of fusion of 196.95 J/g. Furthermore, these microcapsules were characterized in our previous study using a validated LC-MS/MS method for the simultaneous determination of vitexin and isovitexin in rabbit plasma. Our method exhibited similar T_max_ of both formulations, while C_max_ of microencapsulated vitexin and isovitexin were approximately 8-fold and 10-fold higher than that of the crude form, respectively [11].

### 4.2. In Vitro Antioxidant Activity via DPPH Free Radical Scavenging Assay

The DPPH radical scavenging assay was performed using 2,2-diphenyl-1-picrylhydrazyl (DPPH; D4313, Tokyo Chemical Industry Co., Ltd., Tokyo, Japan) according to the method described by Kedare and Singh [92]. The absorbance was measured at 517 nm using a multimode microplate reader (BioTek Synergy HTX, Agilent Technology, Inc., Santa Clara, CA, USA). Each sample was analyzed in triplicate. The result was presented using the IC_50_ value (μg/mL) and compared with a positive control (ascorbic acid).

### 4.3. In Vivo Experimental Design

#### 4.3.1. Animals

Healthy male Swiss albino mice, weighing approximately 20.2 ± 2 g, were provided by the Pasteur Institute in Ho Chi Minh City, Vietnam. Animals were randomized and acclimatized to housing conditions for 7 days prior to the experimental protocols. The mice were fed a standard diet with ad libitum access to water. Mice were kept under controlled conditions at 22 ± 3 °C, relative humidity of 50–60%, and a 12 h light/dark cycle. The experiments were conducted in the Laboratory Animal House, Department of Pharmacology, School of Pharmacy, University of Medicine and Pharmacy at Ho Chi Minh City. All the protocols were approved by the Ethics Committee of the University of Medicine and Pharmacy at Ho Chi Minh City (Decision No. 2035/GCN-HDDDNCTDV). The study was reported with reference to ARRIVE 2.0 guidelines.

#### 4.3.2. Acute and Repeated Restraint Stress Model

**Figure 13:**
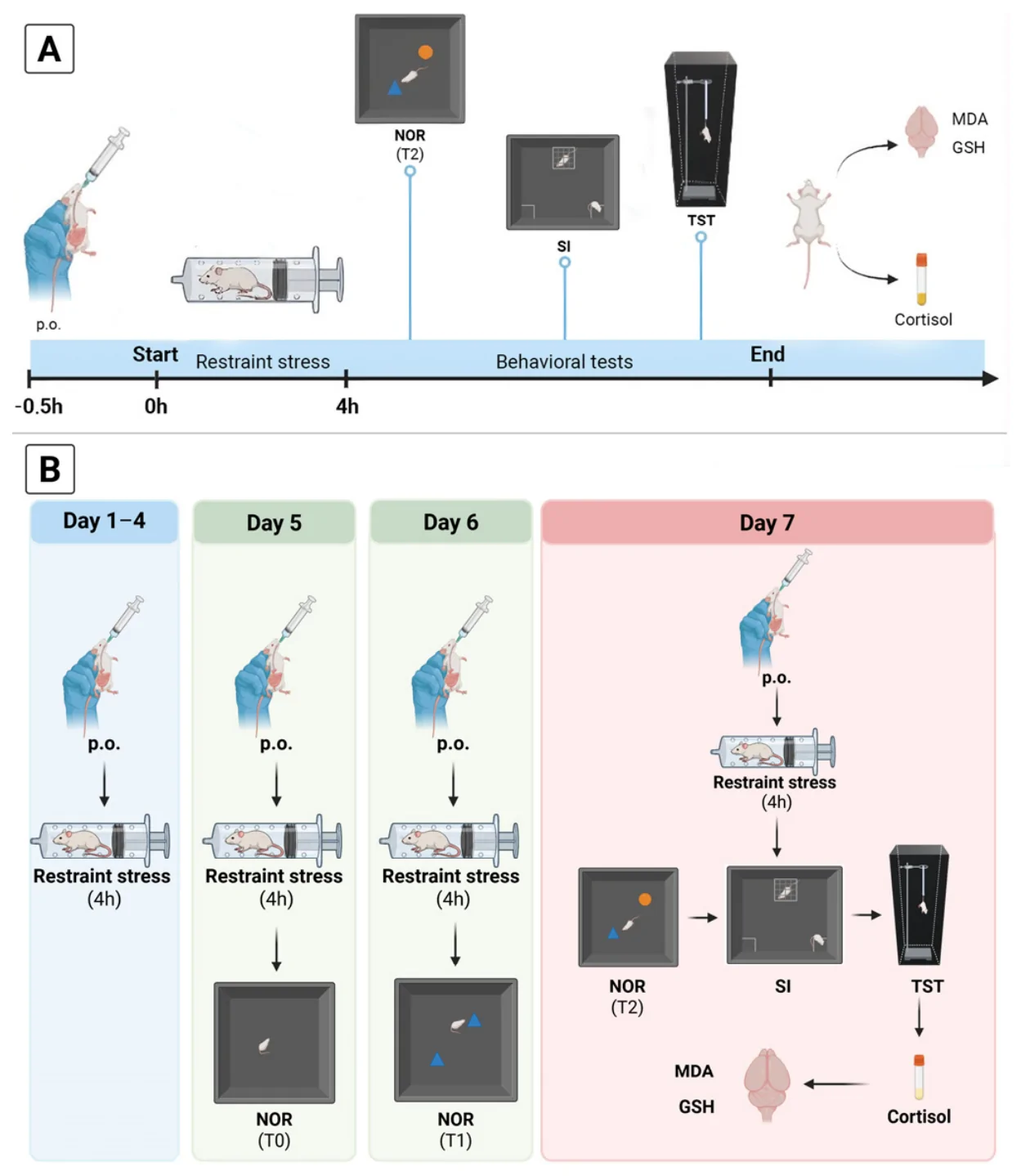
(**A**) Acute model: NOR T0 and T1 were performed before the test day; oral treatment preceded 4 h restraint by 30 min, followed by NOR T2, SI, TST, and blood and brain collection. (**B**) Repeated model: oral treatment preceded 4 h restraint by 30 min daily for 7 days; NOR T0, T1, and T2 were conducted on Days 5, 6, and 7, respectively, followed by SI, TST, and blood and brain collection on Day 7.

Mice received treatments (Table 1) 30 min prior to stress induction. Both the crude and microencapsulated formulations were administered at an equivalent dose of 30 mg/kg, expressed as the total content of active compounds (vitexin and isovitexin). Animals were restrained in ventilated 50 mL syringes for 4 h to restrict movement without complete immobilization. After 4 h, mice underwent the novel object recognition (NOR) testing phase (T2), followed by the social interaction test (SI) and tail suspension test (TST). The preceding NOR habituation (T0) and familiarization (T1) phases were conducted on the two preceding days. Animals from different experimental groups were interleaved during behavioral testing and sample collection to minimize potential order and time-related effects. Blood samples were collected within 90 min after restraint stress for serum cortisol analysis, taking into account the time-dependent glucocorticoid response following restraint stress [58,94]. The hippocampus (HPC), hypothalamus (HYP), and prefrontal cortex (PFC) were subsequently dissected for malondialdehyde (MDA) and glutathione (GSH) assays (Figure 13). All the behavioral tests and biochemical assays were performed under identical conditions by an investigator blinded to group allocation to minimize potential confounding factors. During the experiments, mice whose bodyweight has decreased by more than 20% compared with control animals were humanely sacrificed using cervical dislocation technique according to OECD guidelines.

The restraint stress was applied once in the acute stress model, and the behavioral tests were conducted immediately after. This procedure was repeated once daily for 7 consecutive days at the same time, and the behavioral tests were conducted on day 7 following the final restraint session.

### 4.4. Determination of Malondialdehyde (MDA) and Glutathione (GSH) Levels

#### 4.4.1. Thiobarbituric Acid Reactive Substances (TBARS) Assay

MDA levels were determined using the TBARS assay according to the method described by [93] with slight modifications. Brain homogenized in phosphate buffer (pH 7.5) was precipitated with 10% trichloroacetic acid and centrifuged at 9600 rpm for 15 min at 4 °C. The supernatant was reacted with 0.8% 2-thiobarbituric acid (TBA; T5500, Sigma-Aldrich, St. Louis, MO, USA) at 95 °C for 15 min, and absorbance was measured at 532 nm using a multimode microplate reader (BioTek Synergy HTX, Agilent Technology, Inc., Santa Clara, CA, USA). Each sample was analyzed in triplicate.

#### 4.4.2. GSH Assay

GSH levels were determined with minor modifications according to the method described by Rahman et al. [95]. Briefly, the supernatant was reacted with 5 mM 5,5′-dithiobis(2-nitrobenzoic acid) (DTNB; PI22582, Thermo Fisher Scientific Inc., Loughborough, UK), and absorbance was measured at 412 nm in kinetic mode using a multimode microplate reader (BioTek Synergy HTX, Agilent Technology, Inc., Santa Clara, CA, USA).

#### 4.4.3. Protein Assay

Total protein concentration in the brain homogenate was measured following the instructions provided with the Qubit^TM^ protein assay kits (Q33212, Thermo Fisher Scientific Inc., Waltham, MA, USA). MDA and GSH levels were normalized to the protein concentration and expressed as μmol/mg.

### 4.5. Determination of Serum Cortisol Levels

Serum cortisol assay was conducted using the VIDAS^®^ Cortisol S kit (BioMérieux S.A., Marcy-l’Étoile, France) according to the manufacturer’s guidelines. Results presented in nmol/L for intra-study comparison.

### 4.6. Behavioral Tests

#### 4.6.1. Novel Object Recognition Test (NOR)

**Figure 14:**
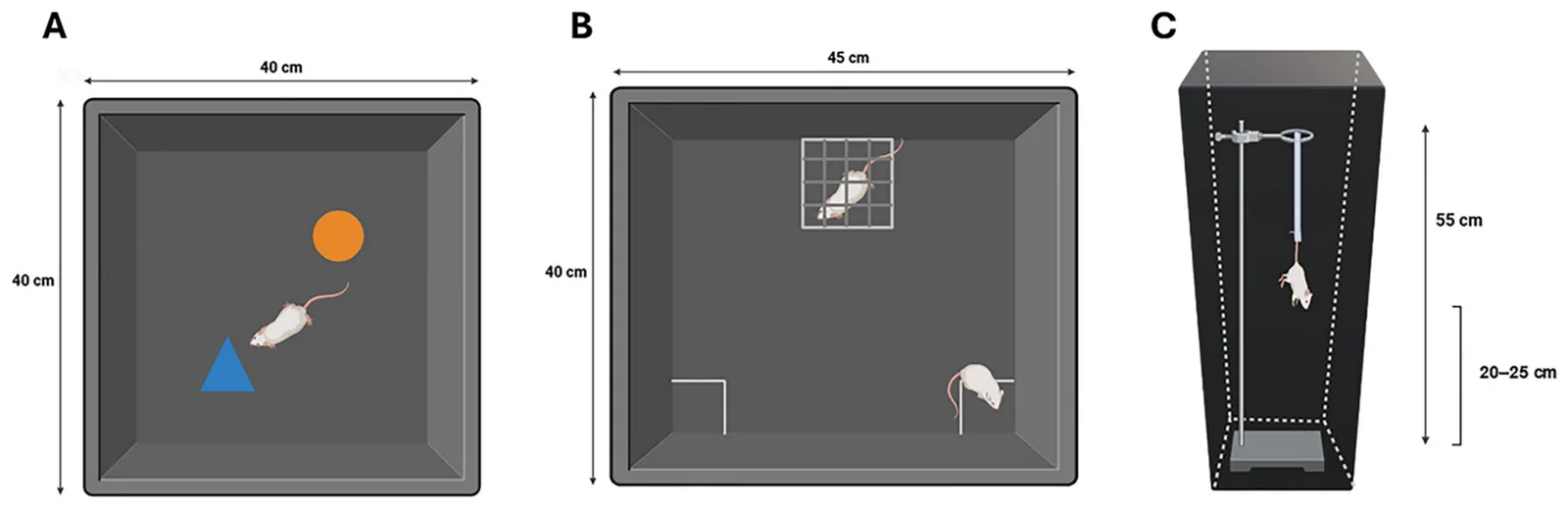
Schematic presentation of behavioral test apparatus. Novel object recognition test—NOR (**A**); social interaction test—SI (**B**); and tail suspension test—TST (**C**). Dimensions are expressed in centimeters.

The novel object recognition apparatus included an open box measuring 40 × 40 × 40 cm (Figure 14). The test consisted of three phases, as described in the protocol reported by Lueptow (2017) [96] and Leger (2013) [97]. For both stress paradigms, habituation (T0), familiarization (T1), and testing (T2) were conducted on Day 2, Day 1, and Day 0 relative to the final behavioral testing day, respectively. In the ARS model, Day 0 corresponded to the single restraint-stress day, whereas in the RRS model, it corresponded to Day 7 of repeated restraint stress. Accordingly, T0 and T1 in the RRS model were performed on Days 5 and 6, respectively.

- Habituation (T0): Each mouse was placed in the center of the empty arena and allowed to explore freely for 5 min.
- Familiarization (T1): Two identical objects were placed in opposite quadrants of the arena. 24 h after T0, each mouse was allowed to explore these objects freely for 5 min.
- Testing (T2): After a retention interval of 24 h, one familiar object was replaced with a novel object, and the mouse was allowed to explore both objects for 10 min.

Mice were excluded when they did not reach a total exploration time of 20 s by 5 min for both objects during T1 and T2. To minimize potential object or spatial preferences, objects were counterbalanced so that each object served equally as the familiar or novel object. The position of the novel object was also counterbalanced across the quadrants of the arena. Object exploration was defined as the mDouse actively directing its nose toward an object within 2–3 cm, accompanied by sniffing or active vibrissae movement; sitting on or climbing the object without active exploration was not counted. The arena and objects were thoroughly cleaned with 70% ethanol between mice during each phase to minimize residual odor.

(1) $$\mathrm{Evaluation}\;\mathrm{criteria}:\;\mathrm{NOR}=\frac{\mathrm{New}\;\mathrm{object}\;\mathrm{exploration}\;\mathrm{time}}{\mathrm{Both}\;\mathrm{old}\;\mathrm{and}\;\mathrm{new}\;\mathrm{objects}\;\mathrm{exploration}\;\mathrm{time}}\times 100\%$$

#### 4.6.2. Social Interaction Test (SI)

The social interaction test was adapted from a previously reported social interaction paradigm [98] and performed in an open box (45 × 40 × 40 cm) containing an interaction zone surrounding a wire-mesh cage with a novel, age- and sex-matched Swiss albino mouse and two corner zones at the opposite side. The test lasted 10 min for each mouse and the apparatus was cleaned with 70% ethanol afterwards before continuing with the next mouse. Animal movements were recorded using a digital camera and the trajectories were subsequently analyzed using ToxTrac software version 1.1.

Evaluation criteria were as follows:

(2) $$\mathrm{Social}\;\mathrm{interaction}\;\mathrm{ratio}\;\left(\%\right)=\frac{\mathrm{Time}\;\mathrm{spent}\;\mathrm{in}\;\mathrm{the}\;\mathrm{interaction}\;\mathrm{zone}}{\mathrm{Time}\;\mathrm{spent}\;\mathrm{outside}\;\mathrm{the}\;\mathrm{interaction}\;\mathrm{zone}}\times 100\%$$

(3) $$\mathrm{Social}\;\mathrm{interaction}\;\mathrm{ratio}\;\left(\%\right)=\frac{\mathrm{Time}\;\mathrm{spent}\;\mathrm{in}\;\mathrm{the}\;\mathrm{interaction}\;\mathrm{zone}}{\mathrm{Time}\;\mathrm{spent}\;\mathrm{outside}\;\mathrm{the}\;\mathrm{interaction}\;\mathrm{zone}}\times 100\%$$

#### 4.6.3. Tail Suspension Test (TST)

The tail suspension test was conducted to assess depressive behaviour in mice with minor modifications from the method described by [99]. Mice were suspended by the tail 20–25 cm above the floor for 6 min and the immobility time was recorded.

Evaluation criteria was immobility time (seconds).

### 4.7. Molecular Docking Study

#### 4.7.1. Target Protein Acquisition and Preparation

The three-dimensional experimental structures of the target proteins were retrieved from the Research Collaboratory for Structural Bioinformatics Protein Data Bank. To explore the predicted structural compatibility of vitexin and isovitexin with selected stress-related molecular targets, five macromolecular structures were selected based on structural resolution and availability of relevant ligand-bound conformations. The molecular docking simulations were executed against the following specific complexes: the X-ray structure of the ts3 human serotonin transporter (SERT) complexed with paroxetine at the central site (PDB ID: 5I6X) [100]; the cryo-EM structure of the befiradol-bound 5-HT1A serotonin receptor coupled with the Gi protein complex (PDB ID: 8PKM) [101]; the cryo-EM structure of the human full-length heteromeric α1β3γ2L GABAA receptor in complex with alprazolam, GABA, and the stabilizing megabody Mb38 (PDB ID: 6HUO) [102]; and the glucocorticoid receptor featuring an extended ligand-binding pocket induced by deacylcortivazol (PDB ID: 3BQD) [103] and the Kelch domain of human Keap1 bound to the small-molecule inhibitor fragment 4-oxo-4H-1-benzopyran-2-carboxylic acid (PDB ID: 5WHO) [89]. These ligand-bound structures were selected to provide defined binding cavities for exploratory assessment of the spatial accommodation of the bulky C-glycoside flavonoids. Initial structural refinement and curation were performed using BIOVIA Discovery Studio Visualizer 2025 and UCSF ChimeraX 1.11 [104]. The experimental structures were processed to remove non-biological artifacts, including crystallographic bulk water molecules, co-solvents, detergent lipids (such as cholesterol and cholesteryl hemisuccinate), and auxiliary stabilizing proteins such as Fab fragments (for 5I6X), scFv16 antibodies (for 8PKM), and Mb38 megabodies (for 6HUO). Redundant oligomeric chains were deleted to reduce computational interference; however, structurally relevant components were retained. Specifically, the sodium and chloride ions associated with the outward-facing state of the 5I6X complex and the co-activator peptide present in the 3BQD structure were preserved. Subsequently, the refined protein models were imported into AutoDock Tools version 1.5.7 for computational parameterization. Polar hydrogen atoms were added for hydrogen-bonding calculations, while non-polar hydrogens were merged into their parent carbon atoms. Partial atomic charges were assigned using the Kollman method. Finally, AutoDock 4 atom types were assigned, and the parameterized macromolecular structures were exported in PDBQT format for subsequent molecular docking simulations [105].

#### 4.7.2. Ligand Acquisition and Parameterization

The chemical structures of the primary investigated phytochemicals, the C-glycosyl flavones vitexin and isovitexin, were retrieved from the PubChem compound database. Conversely, the native co-crystallized reference ligands (paroxetine, befiradol, alprazolam, deacylcortivazol, and the 4-oxo-4H-1-benzopyran-2-carboxylic acid fragment) were directly extracted from their respective experimental holo-complexes using BIOVIA Discovery Studio Visualizer 2025. Extracting these native inhibitors and agonists directly from the structural data rigorously preserves their experimentally validated bioactive conformations, which is fundamentally crucial for the subsequent redocking procedures. To ensure accurate chemical perception and parameterization, all acquired flavonoids and extracted reference ligands were systematically processed using Meeko, which was specifically employed to handle the proper perception of fundamental chemical properties, including covalent bonds, formal charges, and pH-dependent protonation states adjusted to simulate physiological conditions. Furthermore, the Meeko package effectively managed the assignment of active rotatable bonds, granting the necessary conformational flexibility for the bulky C-glycoside appendages of vitexin and isovitexin to structurally adapt within the restricted binding cavities. Following this automated parameterization, Meeko successfully generated the fully optimized ligand structures in the docking-compatible PDBQT format, rendering them ready for the molecular docking pipeline [105].

#### 4.7.3. Grid Box Configuration

The computational search space for the molecular docking simulations was precisely delineated utilizing AutoDock Tools version 1.5.7. To ensure that the conformational search algorithms exclusively explored the biologically validated active sites, a native ligand-centering strategy was systematically employed. Specifically, the geometric center of each grid box was established by extracting the three-dimensional centroid coordinates of the respective co-crystallized reference ligands inherent to the retrieved experimental holo-complexes. The dimensions of the grid boxes were meticulously optimized to completely encompass the native ligands while providing sufficient spatial volume to accommodate the bulky and conformationally flexible C-glycoside moieties of vitexin and isovitexin without inducing artificial steric clashes. Notably, the grid box dimensions for the glucocorticoid receptor were significantly expanded (24 × 36 × 32 Å) to fully encapsulate the uniquely doubled ligand-binding pocket natively induced by the voluminous deacylcortivazol. Similarly, the search space for the serotonin 1A receptor was longitudinally extended (24 × 40 × 24 Å) to account for the elongated binding channel occupied by befiradol. Furthermore, the exhaustiveness parameter, which dictates the thoroughness of the global search algorithm, was strictly set to 16 for the majority of the macromolecular targets to balance computational efficiency and accuracy. However, for the deeply buried orthosteric binding pocket of the 5-HT_1A_ receptor, the exhaustiveness was strategically elevated to 32 to guarantee comprehensive conformational sampling and prevent entrapment in local energy minima. To eliminate stochastic variations inherent to the Iterated Local Search algorithm in AutoDock Vina, a fixed pseudo-random seed (seed = 42) was applied for all docking runs. A uniform grid spacing of 0.375 Å was maintained across all configurations. The precise spatial parameters established for each specific target are comprehensively detailed in Table 2.

Prior to executing the docking of the investigated C-glycosyl flavones, the accuracy and predictive reliability of the established grid box parameters and the AutoDock Vina scoring algorithm were rigorously validated through a comprehensive redocking procedure. The native co-crystallized ligands were extracted from their respective experimental complexes. Following the parameterization protocol described previously to eliminate their initial experimental conformational memory, these reference molecules were redocked into their designated computational search spaces. The structural fidelity and validity of the computational protocol were quantitatively assessed by calculating the Root Mean Square Deviation (RMSD) between the heavy atoms of the computationally predicted optimal poses and their corresponding reference crystallographic or cryo-EM geometries. A stringent threshold of RMSD ≤ 2.0 Å was established as the standard to confirm a successful and accurate binding pose prediction.

#### 4.7.4. Molecular Docking Simulations

Following validation of the computational search parameters, molecular docking of vitexin and isovitexin was performed against the five selected molecular targets. The simulations were performed using AutoDock Vina 1.2.7 [106,107]. Generated poses were ranked according to the AutoDock Vina predicted binding scores, expressed in kilocalories per mole. For each target–ligand pair, the pose with the most favorable predicted score was selected for subsequent structural interaction analysis. These scores were used for comparative in silico interpretation and were not considered direct measures of biological affinity or functional activity.

#### 4.7.5. Post-Docking Analysis and Visualization

Poses exhibiting the most favorable binding affinities were extracted for detailed in silico structural analysis. BIOVIA Discovery Studio Visualizer 2025 was utilized to inspect the three-dimensional intermolecular interactions, evaluating the spatial accommodation, potential steric hindrances, and overall shape complementarity of the bulky carbohydrate appendages of vitexin and isovitexin within the restricted receptor pockets. Furthermore, two-dimensional interaction diagrams were systematically generated to map and characterize the specific non-covalent contacts formed between the respective pharmacophores and the critical amino acid residues of the active sites. This comprehensive mapping meticulously documented a diverse array of stabilizing forces, including conventional and carbon hydrogen bonds, electrostatic associations (such as salt bridges, π-cation, and π-anion interactions), as well as intricate hydrophobic networks encompassing π-π stacked, π-π T-shaped, and π-alkyl/alkyl contacts. The predicted interacting residues and corresponding interaction types identified through this analysis are summarized in Supplementary Table S1.

### 4.8. Statistical Analysis

A sensitivity power analysis was performed to evaluate the statistical adequacy of the sample sizes used in the completed experiments. The analysis was based on a fixed-effects omnibus one-way ANOVA with five independent experimental groups, a significance level of α = 0.05, and a statistical power of 80%.

Data were expressed as mean ± S.D. (standard deviation), S.E.M. (standard error of mean) or median with interquartile range (IQR). Normality and homogeneity of variance were assessed using the Shapiro–Wilk and Levene’s tests, respectively. Normally distributed data with homogeneous variance were analyzed using one-way analysis of variance (ANOVA) followed by Tukey’s post hoc test for comparisons among three or more groups. For data that did not meet the assumption of normality, the Kruskal–Wallis test followed by Dunn’s post hoc test was applied. Differences are significant when *p* < 0.05. IBM SPSS Statistics 27.0 (IBM Corp., Armonk, NY, USA) was used for statistical analyses. GraphPad Prism 9.5 (GraphPad Software Inc., San Diego, CA, USA) was used for graphs plotting.

## 5. Conclusions

In conclusion, acute and repeated restraint stress were associated with behavioral and biochemical changes in mice. VTX-iVTX and mVTX-iVTX produced variable treatment-associated effects across behavioral and redox-related outcomes in the two stress models. Microencapsulation showed effects comparable to the crude formulation in most parameters, with more pronounced effects in selected outcomes, including social interaction under acute stress and increased GSH levels during repeated stress. Microencapsulation showed effects comparable to the crude formulation in most parameters, with more pronounced effects in some outcomes, including social interaction under acute stress and increased GSH levels during repeated stress. To our knowledge, this study is among the first to investigate the neuropharmacological effects of a vitexin–isovitexin combination in a rodent restraint-stress model. The proposed HPA-axis and molecular mechanisms remain to be experimentally validated in future studies.

## Figures and Tables

**Table 1:** Experimental group assignments for acute restraint stress (ARS) and repeated restraint stress (RRS) models.

Group	Treatment	Dose	Route	ARS (*n* = 10)	RRS (*n* = 12)
Normal control	Distilled water	___	p.o.	No stress	No stress
Stress control	Distilled water	___	p.o.	4 h × 1 day	4 h × 7 days
VTX-iVTX	Vitexin–isovitexin	30 mg/kg	p.o.	4 h × 1 day	4 h × 7 days
mVTX-iVTX	Vitexin–isovitexin-loaded microcapsules	30 mg/kg	p.o.	4 h × 1 day	4 h × 7 days
Fluoxetine	Fluoxetine	25 mg/kg	p.o.	4 h × 1 day	4 h × 7 days

**Table 2:** Grid box parameters established for the selected molecular targets.

Target Protein	PDB ID	Co-Crystallized Ligand	Center X	Center Y	Center Z	Dimensions (Å)	Exhaustiveness
SERT	5I6X	Paroxetine	−32.300	−21.108	2.119	24 × 24 × 24	16
5-HT_1A_	8PKM	Befiradol	108.981	118.548	94.707	24 × 40 × 24	32
GABA_A_	6HUO	Alprazolam	153.961	162.783	161.361	24 × 24 × 24	16
GR	3BQD	Deacylcortivazol	40.837	30.985	9.613	24 × 36 × 32	16
Keap1 (Kelch)	5WHO	Chromone fragment	84.263	38.123	66.392	24 × 24 × 24	16

## Data Availability

The data presented in this study are available upon reasonable request from the corresponding author.

## Supplementary Materials

The following supporting information can be downloaded at https://www.mdpi.com/article/10.3390/ijms27198731/s1.

## Abbreviations

The following abbreviations are used in this manuscript:

WHO	World Health Organization
5-HT	5-hydroxytryptamine
HPA	Hypothalamic–pituitary–adrenal
SSRIs	Selective serotonin reuptake inhibitors
TCAs	Tricyclic antidepressants
MAOIs	Monoamine oxidase inhibitors
CUMS	Chronic unpredictable mild stress
SDS	Social defeat stress
ARS	Acute restraint stress
RRS	Repeated restraint stress
MDA	Malondialdehyde
GSH	Glutathione
NOR	Novel object recognition
Nrf2	Nuclear Factor Erythroid 2 Like 2
SI	Social interaction
TST	Tail suspension test
SERT	Serotonin transporter
HPC	Hippocampus
HYP	Hypothalamus
GR	Glucocorticoid receptor
CRH	Corticotropin-releasing hormone
GABA_A_	Type-A γ-aminobutyric acid
Keap1	Kelch-like ECH-associated protein 1
DSC	Differential scanning calorimetry
DPPH	2,2-diphenyl-1-picrylhydrazyl
TBA	2-thiobarbituric acid
DTNB	5,5′-dithiobis(2-nitrobenzoic acid)
PFC	Prefrontal cortex
VTX-iVTX	Vitexin–isovitexin
mVTX-iVTX	Vitexin–isovitexin-loaded microcapsules
RMSD	Root mean square deviation
S.D.	Standard deviation
S.E.M.	Standard error of mean
IQR	Interquartile range
