# Supplementary material for: Behavioral and Biochemical Effects of Vitexin–Isovitexin-Loaded Microcapsule Combination on Restraint Stress Mouse Model
Source: Int J Mol Sci. 2026 Sep 30;27(19):8731. doi: 10.3390/ijms27198731 (PMC13665335; doi:10.3390/ijms27198731)
Supplement: Supplementary file 1 [file ijms-27-08731-s001.zip › ijms-4528489-supplementary.pdf]

SUPPLEMENTARY INFORMATION

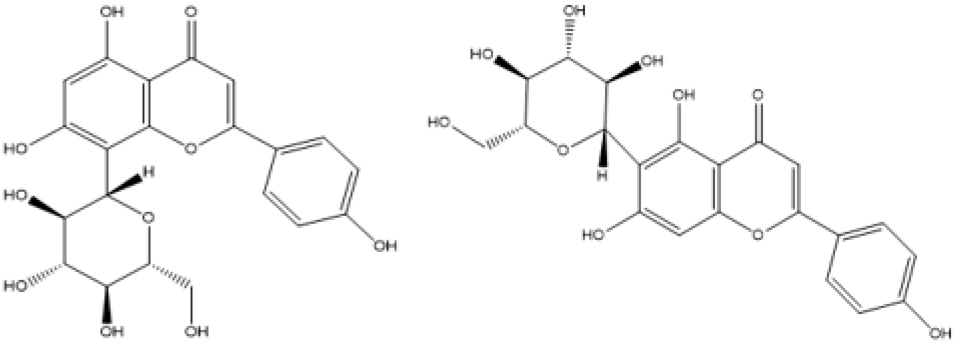

Fig. S1. The structure of vitexin (left) and isovitexin (right)

SUPPLEMENTARY METHODS

Post-docking analysis and visualization

Poses exhibiting the most favorable binding affinities were extracted for detailed *in silico* structural analysis. BIOVIA Discovery Studio Visualizer 2025 was utilized to inspect the three-dimensional intermolecular interactions, evaluating the spatial accommodation, potential steric hindrances, and overall shape complementarity of the bulky carbohydrate appendages of vitexin and isovitexin within the restricted receptor pockets. Furthermore, two-dimensional interaction diagrams were systematically generated to map and characterize the specific non-covalent contacts formed between the respective pharmacophores and the critical amino acid residues of the active sites. This comprehensive mapping meticulously documented a diverse array of stabilizing forces, including conventional and carbon hydrogen bonds, electrostatic associations (such as salt bridges, π-cation, and π-anion interactions), as well as intricate hydrophobic networks encompassing π−π stacked, π−π T-shaped, and π-alkyl/alkyl contacts. Through this rigorous visualization protocol, the structural rationale underlying the competitive inhibition and receptor modulation by the C-glycosyl flavones was profoundly established.

Table S1. Predicted molecular interactions of vitexin and isovitexin with selected protein targets based on molecular docking analysis.

Target	Compound	Residues	Interaction type	Structural interpretation
SERT	Vitexin	Phe335, Tyr95, Ser438, Asp98<br>Asp98, Glu493	hydrogen bonds<br>π-anion<br>π-alkyl	Predicted interactions

		Ile172		within central binding site
	Isovitexin	Phe335, Ser439, Glu493<br>Asp98<br>Tyr176<br>Ile172	hydrogen bonds<br>π-anion<br>π−π T-shaped<br>π-alkyl	
5-HT_{1A}	Vitexin	Ile189, Ser199<br>Lys191<br>Ile189, Ala365	hydrogen bonds<br>π-cation<br>π-alkyl	Potential interactions in orthosteric site
	Isovitexin	Asn386, Lys191<br>Trp102<br>Met92, Ala93<br>Ala93, Cys187	hydrogen bonds<br>π−π T-shaped<br>amide-π stacking<br>π-alkyl	
$GABA_A$	Vitexin	Tyr58, His102, Ser159, Tyr160<br>Ser205<br>Phe77, Tyr210, Tyr160<br>Ala79	hydrogen bonds<br>π-donor hydrogen bond<br>π−π stacked<br>π-alkyl	Predicted interactions within benzodiazepine site
	Isovitexin	Lys156, Asp192, Tyr160, Tyr210<br>Phe77, Tyr160, Tyr210, His102<br>Val203	hydrogen bonds<br>π−π stacked<br>π-alkyl	
Glucocorticoid receptor	Vitexin	Arg611, Glu540, Tyr663, Arg614<br>Val543, Arg611, Arg614	hydrogen bonds<br>π-alkyl	Predicted interactions in expanded binding cavity
	Isovitexin	Gly567<br>Phe623<br>Ala607, Leu608<br>Met604, Leu608, Ala607<br>Met604	hydrogen bonds<br>π−π T-shaped<br>amide-π stacking<br>π-alkyl<br>π-sulfur	
Keap1	Vitexin	Asn382, Ser602, Arg483, Gly433<br>Arg415<br>Ile461	hydrogen bonds<br>electrostatic π-cation<br>π-alkyl	Predicted interactions within Nrf2 binding pocket

SUPPLEMENTARY RESULTS

Redocking validation and detailed interaction networks across target proteins

Prior to executing the docking of the investigated C-glycosyl flavones, the accuracy and predictive reliability of the established grid box parameters and the AutoDock Vina scoring algorithm were rigorously validated through a comprehensive redocking procedure. The native co-crystallized ligands were extracted from their respective experimental complexes. Following the parameterization protocol described previously to eliminate their initial experimental conformational memory, these reference molecules were redocked into their designated computational search spaces.

Table S2. Molecular docking protocol validation parameters via re-docking co-crystallized ligands into the active sites of targets

Target protein	PDB ID	Co-crystallized ligand	Binding energy (kcal/mol)	RMSD (Å)
SERT	5I6X	Paroxetine	-10.87	0.53
5-HT_{1A}	8PKM	Befiradol	-10.89	0.34
$GABA_A$	6HUO	Alprazolam	-11.44	0.67
GR	3BQD	Deacylcortivazol	-14.10	0.54
Keap1 (Kelch)	5WHO	Chromone fragment	-7.32	0.77

Serotonin transporter (SERT)

Redocking of paroxetine yielded a low RMSD (0.53 Å) and potent binding energy (-10.87 kcal/mol). Its piperidine nitrogen formed electrostatic contacts (salt bridge/attractive charge with Asp98; attractive charge with Ser336; π-cation with Tyr95) and a conventional hydrogen bond with Tyr95 (1.90 Å). Additional stabilization occurred via hydrogen bonds with Ala96 (3.16 Å), Ser336 (3.47 Å, carbon H-bond), and Ala169 (3.50 Å, via 1,3-benzodioxole). The aromatic rings established extensive hydrophobic networks, including π−π T-shaped (Phe341, Tyr176), amide-π stacked (Ser438, Ser439), and π-alkyl contacts (Ile172, Ala169, Ala173).

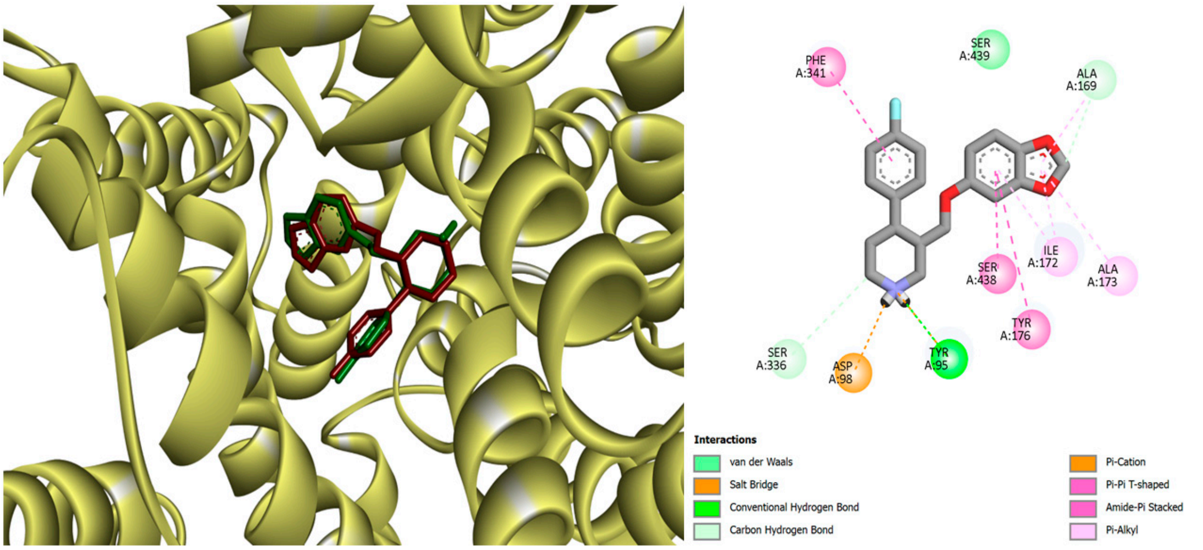

Fig. S2. Redocking paroxetine (red: co-crystalline molecule, green: redock molecule) poses and 2D interaction diagram of paroxetine with the human serotonin transporter

Serotonin 1A receptor (5−HT_{1A})

Redocking of befiradol into the 5−HT_{1A} receptor validated the protocol, yielding a highly accurate RMSD of 0.34 Å and a robust binding affinity of -10.89 kcal/mol. The ligand is effectively anchored by its secondary amine (-NH-) linker, which forms a critical salt bridge and electrostatic contact with Asp116. This stability is heavily reinforced by an extensive hydrophobic scaffold: the 5-methylpyridine moiety engages in π−π T-shaped stacking with Phe362 and alkyl contacts with Ala203, Val117, and Ile167; the 3-chlorophenyl ring participates in amide-π stacking with Met92 and Ala93; and the central piperidine forms a π-alkyl interaction with Tyr390.

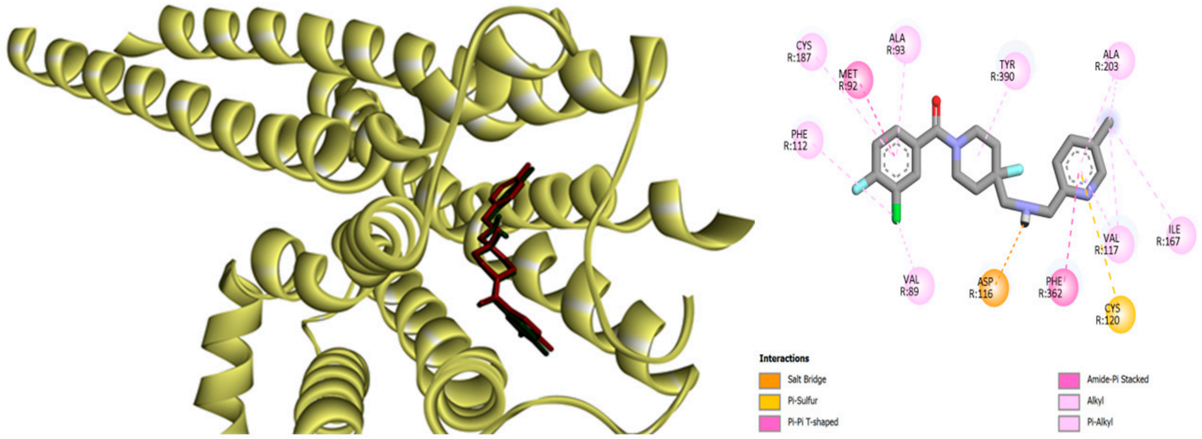

Fig. S3. Redocking befiradol (red: co-crystalline molecule, green: redock molecule) poses and 2D interaction diagram of befiradol with the serotonin 5-HT_{1A} receptor

GABA$_A$ receptor

The robustness of the docking parameters at the benzodiazepine site was evidenced by the successful reproduction of the alprazolam-GABA$_A$ complex. Computational redocking accurately mirrored the experimental cryo-EM geometry, as demonstrated by a low RMSD (0.668 Å) and a strong binding energy of -11.44 kcal/mol. The ligand established a conventional hydrogen bond (2.66 Å, diazepine azomethine N) and a carbon hydrogen bond (2.88 Å, triazole N) with Ser205. Its benzene chlorine atom formed a critical halogen interaction with His102, alongside π-alkyl contacts with Phe77 and His102. An extensive hydrophobic network included π−π stacking with Tyr210 (pendant phenyl) and Tyr58 (triazole), plus π−π T-shaped contacts with Phe100 and Tyr160 (pendant phenyl).

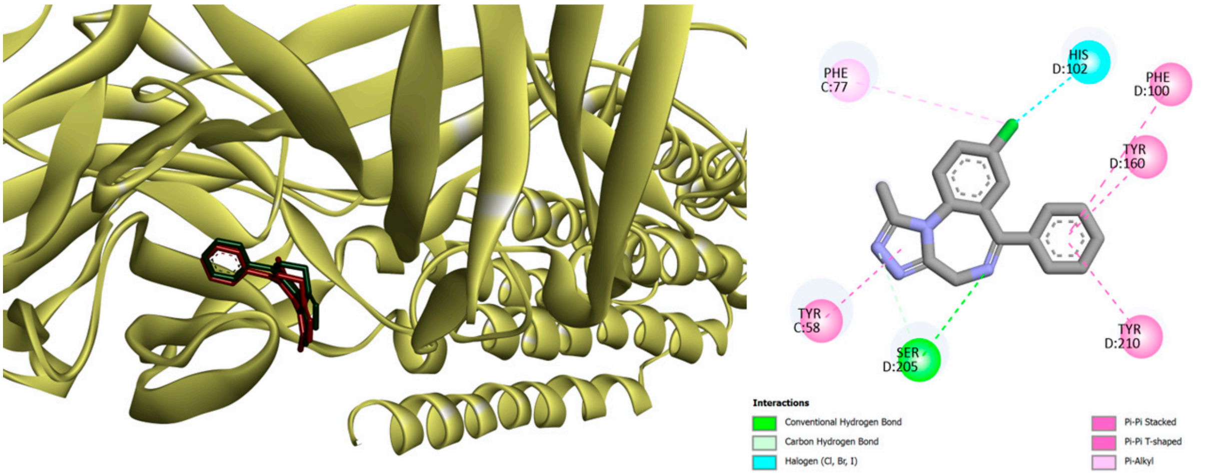

Fig. S4. Redocking alprazolam (red: co-crystalline molecule, green: redock molecule) poses and 2D interaction diagram of alprazolam with the GABAA receptor

Glucocorticoid receptor

The predicted pose for deacylcortivazol achieved a significant binding affinity of -14.1 kcal/mol and a minimal RMSD of 0.538 Å. Its signature hydroxyketone moiety (steroid C1-OH and acetonyl OH) formed dual conventional hydrogen bonds with Asn564 (2.66 and 2.77 Å), alongside a carbon hydrogen bond between the C20-carbonyl and Cys736 (2.45 Å). The phenyl ring engaged in amide-π stacking (Ala607, Leu608), π-alkyl contacts (Ala607, Ala608), and a distinctive π-sulfur interaction with Met604. Additionally, the steroid C5-methyl group provided peripheral stability via alkyl contacts with Ala605, Leu608, and Met646.

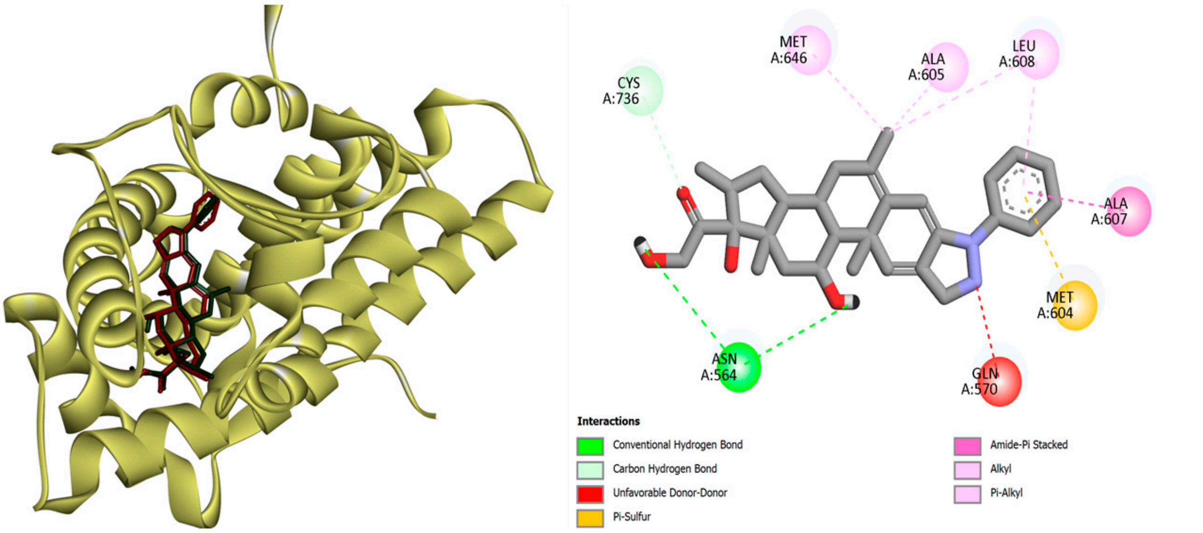

Fig. S5. Redocking deacylcortivazol (red: co-crystalline molecule, green: redock molecule) poses and 2D interaction diagram of deacylcortivazol with the glucocorticoid receptor

Keap1 domain

The Keap1 docking framework was validated by redocking the 4-oxo-4H-1-benzopyran-2-carboxylic acid fragment into the Kelch domain. The computational model demonstrated its predictive robustness by yielding a minimal RMSD (0.768 Å) and a favorable score of -7.323 kcal/mol, thereby establishing a validated baseline for investigating homologous flavone scaffolds. The ligand established conventional hydrogen bonds with Arg483 (2.01 Å, carboxylic carbonyl; 2.19 Å, gamma-pyrone oxygen) and a carbon hydrogen bond with Ser555 (2.92 Å, C-4 ketone). Its carboxylic group acted as a critical anchor, forming a salt bridge (Arg415), attractive charges (Arg415, Arg483), and a π-anion contact (Phe478, via hydroxyl). The planar core was heavily stabilized by π−π stacked interactions with Tyr525 involving both the benzene (A) and gamma-pyrone (B) rings.

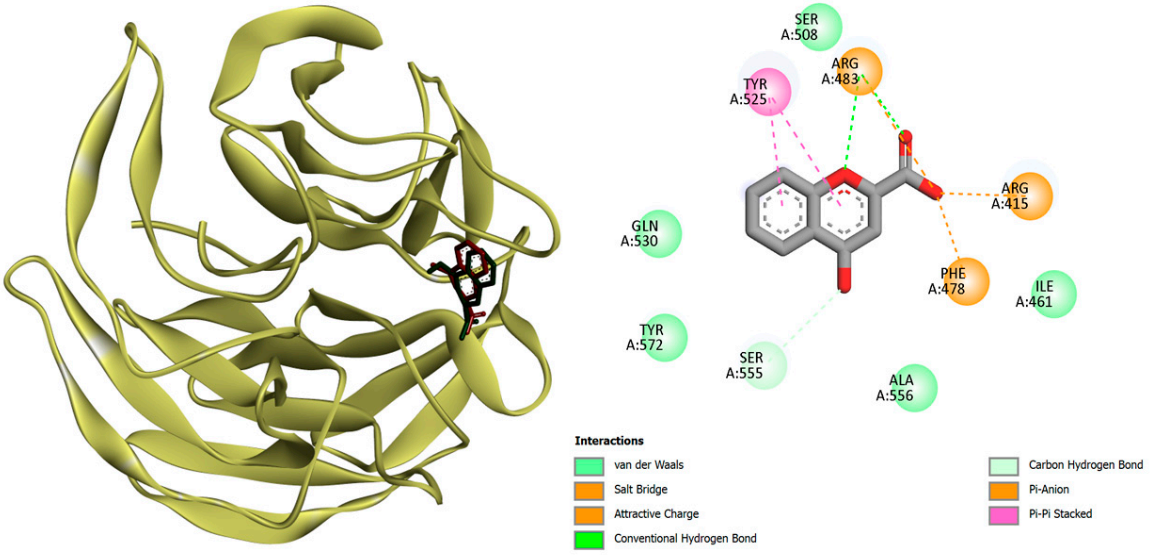

Fig. S6. Redocking 4-oxo-4H-1-benzopyran-2-carboxylic acid (red: co-crystalline molecule, green: redock molecule) poses and 2D interaction diagram of ligand with the Keap1 domain.
